# Microbial planktonic communities in the Red Sea: high levels of spatial and temporal variability shaped by nutrient availability and turbulence

**DOI:** 10.1038/s41598-017-06928-z

**Published:** 2017-07-26

**Authors:** John K. Pearman, Joanne Ellis, Xabier Irigoien, Y. V. B. Sarma, Burton H. Jones, Susana Carvalho

**Affiliations:** 1 King Abdullah University of Science and Technology (KAUST), Red Sea Research Center (RSRC), Biological and Environmental Sciences and Engineering (BESE), Thuwal, 23955-6900 Saudi Arabia; 2 AZTI tecnalia, Herrera Kaia, Portualedea z/g Pasaia, Gipuzkoa, 20110 Spain

## Abstract

The semi-enclosed nature of the Red Sea (20.2°N–38.5°N) makes it a natural laboratory to study the influence of environmental gradients on microbial communities. This study investigates the composition and structure of microbial prokaryotes and eukaryotes using molecular methods, targeting ribosomal RNA genes across different regions and seasons. The interaction between spatial and temporal scales results in different scenarios of turbulence and nutrient conditions allowing for testing of ecological theory that categorizes the response of the plankton community to these variations. The prokaryotic reads are mainly comprised of Cyanobacteria and Proteobacteria (Alpha and Gamma), with eukaryotic reads dominated by Dinophyceae and Syndiniophyceae. Periodic increases in the proportion of Mamiellophyceae and Bacillariophyceae reads were associated with alterations in the physical oceanography leading to nutrient increases either through the influx of Gulf of Aden Intermediate Water (south in the fall) or through water column mixing processes (north in the spring). We observed that in general dissimilarity amongst microbial communities increased when nutrient concentrations were higher, whereas richness (observed OTUs) was higher in scenarios of higher turbulence. Maximum abundance models showed the differential responses of dominant taxa to temperature giving an indication how taxa will respond as waters become warmer and more oligotrophic.

## Introduction

Ecological and biogeochemical processes in the ocean are dependent on a diverse assemblage of microbes including members from Archaea, Bacteria and Eukarya. The diverse plankton assemblages comprising both prokaryotes and eukaryotes fulfill a wide variety of ecological roles in the marine system including carbon fixation (e.g., refs 1 and 2), biogeochemical cycling^1, 3^ and trophic energy transfer (e.g., ref. 4). Approximately half of the global primary production is carried out by oceanic microbes^5^, with contributions from the bacterial genera *Synechococcus* and *Prochlorococcus*, and diverse lineages of small eukaryotes, being especially important^2^. In the oligotrophic regions, this primary production is tightly recycled in the microbial loop, with a small proportion being transferred into higher trophic levels^4^.

Until the regular use of molecular techniques within the marine environment, studies generally relied on morphological characteristics (limiting investigations to those taxa which could be identified under a microscope) or through pigment analysis (limited to pigmented taxa). Therefore, until relatively recently, a detailed understanding of the biological processes occurring in the marine environment was impossible and an integrated assessment of diversity was missing. Indeed, the full extent of their diversity, especially among the eukaryotes, is still poorly understood with novel clades, even at the Kingdom level of classification, being discovered relatively recently (i.e., Picobiliphyta^6^ or Rappemonada^7^). Molecular techniques have been used to examine changes in the community of marine plankton across spatial (e.g., refs 8–10) and temporal (e.g., ref. 11) gradients and to assess the effects of environmental patterns on their composition. In the Red Sea, the number of molecular studies investigating the diversity of the microbial component is extremely limited (e.g., refs 12–16). Other methods have investigated the planktonic diversity in the Red Sea including fingerprinting techniques^17^, microscopy and HPLC^18^. Studies in the Red Sea tend to be restricted in their extent either spatially or temporally. For example, studies undertaken by Kürten *et al*.^17^, Pearman *et al*.^16^ and Kheireddine *et al*.^19^ have a wide spatial coverage but are limited to a single temporal period while Touliabah *et al*.^20^ and Al-Najjar *et al*.^18^ investigated seasonal effects in planktonic communities in a restricted geographic range.

Understanding the control mechanisms and resilience of communities is a critical topic in ecology, especially considering the ever increasing climatic and anthropogenic impacts the marine environment is subjected to. Physical processes can determine the predominant planktonic groups present in a pelagic environment and inherent patterns in size frequency distribution, size spectra and the community composition that are prevalent^21^. For example, it has been shown that the size structure of the phytoplankton community shows a relatively stable inter-annual trend in lake systems^21^ with Gin *et al*.^22^ showing less variability in oligotrophic marine waters compared with coastal waters. Here, stability is not suggested as being a constant but more oscillations around a central point with a known periodicity (i.e., seasonal cycles)^21^. Margalef^23^ and expanded on by Cullen *et al*.^24^ proposed that a combination of turbulence (descried as turbulent mixing of the water column by external forces, such as winds, tides, or upwelling) and nutrient concentrations, which can be affected by seasonal patterns, could determine the community structure. This concept has been further expanded to incorporate further effects or response traits to explain community structures in the plankton^25^.

It has been proposed that in low nutrient and low turbulence areas, typical of stratified tropical oceans the competition for nutrients and the retention of these nutrients, via recycling of organic nutrients, within the microbial loop is the dominant process resulting in a predominance of picophytoplankton (e.g., *Synechococcus*, *Prochlorococcus* and eukaryotic phytoplankton less than 2 μm) and slow growing groups with specialist strategies (e.g., mixotrophy)^24, 25^. Indeed, it has been found that in a stable stratified water column, oscillations in productivity^26^, abundance^27, 28^ and community structure^29^ are minor. As nutrients increase within the system it is proposed there is an increase in biomass and the size of cells but with a slower turnover^24^. It was further proposed that as turbulence increased toward a high nutrient and turbulence situation increased size and biomass would be favored, and there would be transient selection for taxa with rapid growth (e.g., diatoms)^24, 25^. One further category was proposed by Cullen *et al*.^24^ that of the high turbulence and low nutrient area where low biomass and turnover would be prevalent with a selection for organisms, which efficiently used light and nutrients.

In order to test whether responses of plankton in the Red Sea conform to current morphologic-based ecological theories we tested responses over large spatial and temporal gradients using molecular data. The Red Sea is a narrow, semi-confined basin, with limited exchange to other seas. Distinct gradients in temperature, salinity and nutrients are observed along its latitudinal axis. Temperature increases from north to south, salinity generally follows the opposite trend^30^, and nutrients are higher in southern regions compared with the north^13, 16, 31^. Satellite imaging in the main body of the Red Sea indicates a strong seasonality in surface chlorophyll *a* concentrations^32^. During the Arabian Sea northeast monsoon (winter), the prevailing winds over the Red Sea are SE to SSE in the south resulting in a two-layer exchange of water across the Bab el Mandab region^33^. The winds bring surface water into the Red Sea from the Gulf of Aden while Red Sea deep-water outflows into the Gulf^34^. In the northern Red Sea, vertical mixing of the water column can bring nutrients to the surface allowing for increased chlorophyll *a* concentrations^35^. During the Arabian Sea southwest monsoon (summer), the wind forcing is from the NW along the entire Red Sea. The shift to NW winds in the south leads to a three-layer flow pattern with the surface water being driven from the Red Sea into the Gulf of Aden, while the deep water continues to be exported from the Red Sea. The Gulf of Aden Intermediate Water (GAIW), which can occupy up to 70% of the water column, transports cool, lower salinity, nutrient-rich water into the southern Red Sea contributing to the productivity of the system. Despite the increasing body of literature focusing on the Red Sea circulation patterns, a better understanding of how they can affect the biogeochemical processes is still needed. Further, due to the effects of global warming a larger proportion of the world’s oceans is likely to become warmer and more oligotrophic. This makes the Red Sea, which has a gradient in its upper layer from lower temperature and low nutrients in the north to high temperature and higher nutrients in the south, a perfect natural laboratory to study the structure and diversity of microbial plankton communities and their main environmental drivers. Seasonal changes in not only circulation patterns and consequent alterations of water turbulence and nutrient availability but also temperature and salinity allow the investigation of the responses of microbial communities to alterations in prevailing environmental variables under different scenarios.

In accordance with the categories proposed by Cullen *et al*.^24^, we propose that the food web in the northern region of the Red Sea will be dominated by the microbial loop in the summer, while the more nutrient rich southern region will show an increase in the abundance of larger cells typical of fast growing taxa (e.g., diatoms). We do anticipate intra-region seasonal variability driven by changes in temperature and nutrient availability. We undertake species distribution modeling of some of the dominant taxa observed in the Red Sea to gain a greater understanding of the response of these groups to changes in their environment and so increasing the understanding of the limits to their distribution that can be of interest when addressing ecological trajectories under climate change scenarios.

## Results

### Physical structure

**Figure 1:**
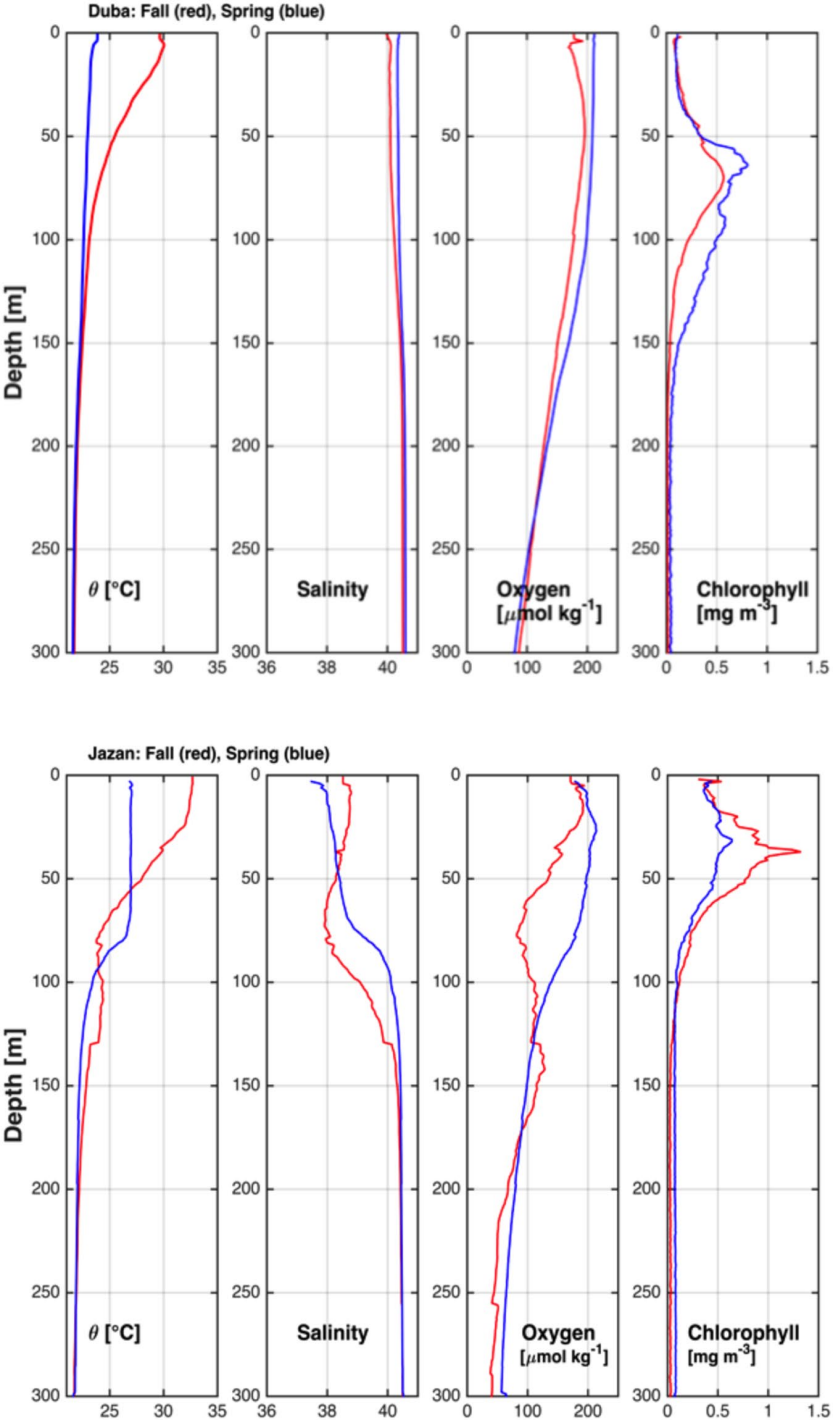
Mean vertical profiles of the hydrographic properties in the northern Red Sea (above) and the southern Red Sea (below).

Mean profiles in the north in spring showed a predominantly mixed water column (mixed layer depth 84 m) with average chlorophyll maximum at approximately 70 m (Fig. 1) (Supplementary Table 1). In the fall, the mixed layer is considerably shallower at 10 m. In the south, the upper layer shows strong seasonality with average chlorophyll maximum at about 40 m. The mixed layer was approximately 50 m in the spring but only 15 m in the fall (Fig. 1) (Supplementary Table 1). In the south, an intrusion of low temperature, low salinity and low oxygen water is observed between 50 and 100 m depth in the water column during fall.

### Biological data

After quality checking of the sequencing reads there were 9442538 prokaryotic reads and 17665062 eukaryotic reads and these reads were used for the clustering as described in the methods. After reference sequences had been taxonomically assigned, OTUs not taxonomically relevant to the current study were removed (Eukaryotes: non-identified phyla or metazoan, 1808 OTUs; Prokaryotes: non-identified phyla or chloroplasts, 1653 OTUs). After multiple rarefactions at an even depth this resulted in 4211 (prokaryotes) and 3054 (eukaryotes) OTUs across all regions and both seasons.

There were no cosmopolitan eukaryotic OTUs with only 36 OTUs (1.2%) being observed in >90% of the samples. 1453 OTUs (47.6%) were present in less than 10% of the samples. In contrast, the prokaryotic component presented 11 OTUs (0.3%), which were observed in all samples, with 77 (1.8%) occurring in at least 90% of the samples. Similar to the eukaryotes, a substantially higher proportion of OTUs were found in fewer than 10% of samples (1497 OTUs, 35.5%). Only 477 prokaryotic (11.3%) and 518 eukaryotic (17.0%) OTUs were shared between all regions/depths/season. For both the eukaryotic and prokaryotic components the central region had a lower number of shared OTUs with either northern or southern regions than those extreme regions did with each other (Supplementary Figure 1).

### Large-scale patterns of variability

#### Regional variability – three regions, spring period

Prokaryotic and eukaryotic fractions varied significantly among regions in terms of Faith’s diversity (PD) (ANOVA; F = 10.374; p < 0.001 and F = 5.545; p = 0.006, respectively). Faith’s diversity of prokaryotes also changed significantly with “Depth” (F = 4.411; p = 0.041). Post-hoc tests (Tukey HSD) revealed that the southern region had the highest diversity with the lowest observed in the central region.

#### Seasonal variability – north and south regions, spring and fall periods

The prokaryotic fraction had a significantly different Faith’s diversity across “Region” (F = 6.492; p = 0.0121), “Depth” (F = 32.158; p < 0.001) and “Season” (F = 9.758; p = 0.002). For the eukaryotic fraction, differences across regions and season were inconsistent, i.e., there was a significant interaction between “Region” and “Season” (F = 6.932; p = 0.010). Post-hoc tests revealed that sampling in the north in the fall had significantly higher values of Faith’s diversity than either region in the spring as well as the south in the fall.

Four scenarios (North_Fall, North_Spring, South_Fall and South_Spring) were established based on turbulence and nutrient conditions. In general, higher nitrate concentrations are registered in the South_Fall whilst the South_Spring had the highest levels of phosphate and silicate (Supplementary Figure 2). In terms of community dissimilarity (measured with unweighted and weighted UniFrac), higher levels of dissimilarity were observed in the South_Fall (weighted UniFrac) and South_Spring (unweighted UniFrac) (Supplementary Table 2 and Supplementary Figure 2). A similar trend was observed for the prokaryote structure (unweighted UniFrac) where both the South_Spring and South_Fall (no significant difference between the two) were higher than either the North_Spring or North_Fall.

### Variability in taxonomic groups

**Figure 2:**
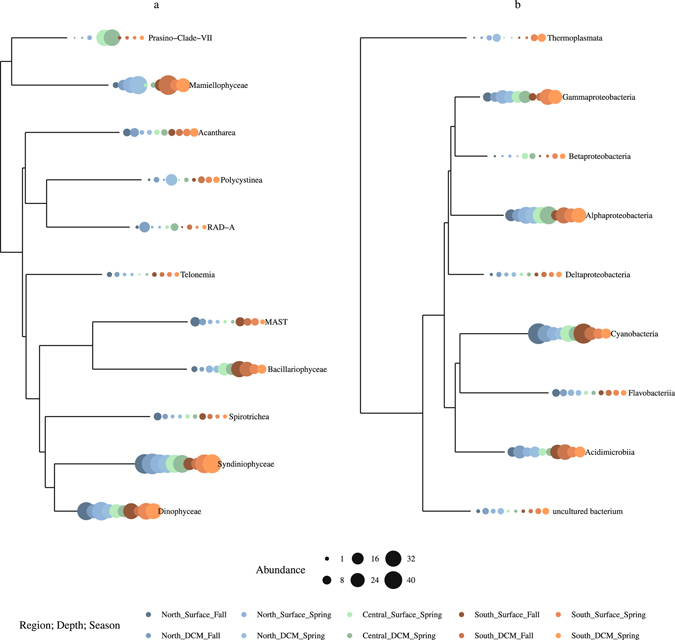
Phylogenetic tree of the main classes of (**a**) eukaryotes and (**b**) prokaryotes based on alignments of the 18 S rRNA and 16 S rRNA genes, respectively. The proportional abundance of specific classes in each regions (north, south), depths (surface, deep chlorophyll maximum – DCM), and seasons (spring, fall) (color coded) is denoted by the size of the circle at the end of each branch.

The main dominant eukaryotic groups in the Red Sea in both seasons were the Alveolata classes Dinophyceae and Syndiniophyceae (mainly group I clade 1, 4 and 5 and group II clade 6, 7 and 10+11) with an average percentage of eukaryotic reads of 20.2% and 28.7%, respectively (Fig. 2a). Bacillariophyta increased in dominance in the south during the fall, reaching a peak of 23.7% of total number of reads. The eukaryotes Mamiellophyceae also showed increased numbers of reads in the southern region during the fall (average of 36.1% of reads at the Deep Chlorophyll Maximum (DCM)) and in the north during the spring (average of 30.5% at the DCM). The three main Mamiellophyceae genera (*Ostreococcus*, *Micromonas* and *Bathycoccus*) showed distinct patterns of distribution: *Ostreococcus* was more prevalent in the spring in the north while *Micromonas* and *Bathycoccus* presented higher proportional abundances in the south in the fall (Supplementary Figure 3). The Prasinophyte-Clade VII only had a high number of reads in the central region during the spring.

In terms of the prokaryotes, only four classes accounted for on average 87% of the reads: Cyanobacteria (28.6%), Alphaproteobacteria (27.8%), Gammaproteobacteria (16%), and Acidimicrobiia (14.6%) (Fig. 2b). Among the Cyanobacteria, *Synechococcus*, and to a lesser extent *Prochlococcus*, were the dominant genera (Supplementary Figure 4). SAR11 and SAR86 were the main contributors of Alphaproteobacteria and Gammaproteobacteria, respectively. Finally, the Acidimicrobiia mainly consisted of clade OM1. Although never accounting for a high number of reads, the archaeal class Thermoplasmata showed increased abundances in the spring period in both the northern and southern regions.

**Figure 3:**
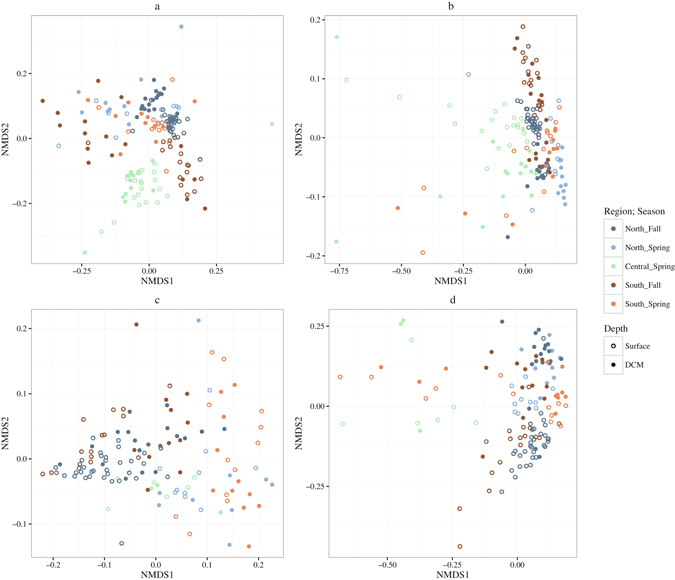
NMDS for eukaryotes – (**a**) weighted UniFrac (**b**) unweighted UniFrac and prokaryotes (**c**) weighted UniFrac and (**d**) unweighted UniFrac.

Non-metric multidimensional scaling (NMDS) indicated that both the structure (weighted) and composition (unweighted) of the eukaryotic and prokaryotic communities changed according to region, season and depth (Fig. 3). For the eukaryotes, both unweighted and weighted, significant interactions were observed between the factors, suggesting inconsistent variations in the trends. Pairwise comparisons (Supplementary Table 3) showed that, in general, community composition differed significantly with region and season. In general the surface community was also different from that at the DCM, with exception of those stations in the south. Community structure of eukaryotes showed similar patterns for the region and season. For the depths it was found that in the spring the surface and DCM were similar, differing in the fall. Interactions were also observed in both the weighted and unweighted matrices for prokaryotes. Pairwise, comparisons showed general significant differences between the north and the south, seasons and depth (Supplementary Table 3).

**Figure 4:**
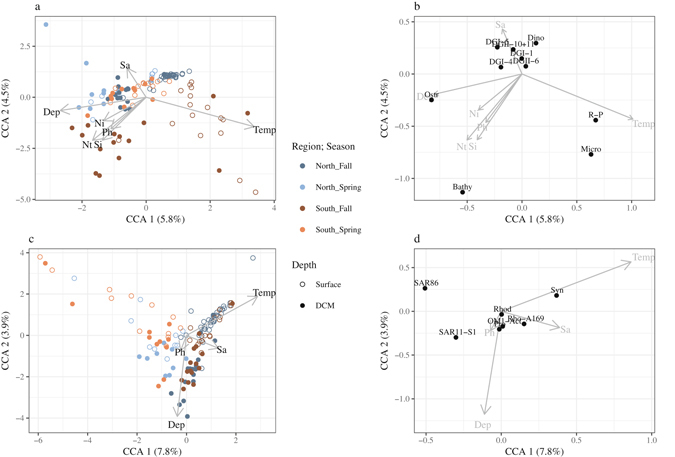
CCA analysis of the eukaryotic stations (**a**) and most abundant eukaryotic groups (**b**) and for the prokaryote stations (**c**) and the prokaryotic groups (**d**) with the environmental data. The seasons and regions are color coded while open circles denote samples taken at the surface with closed circles denoting the DCM. For (**b**) the taxa codes are: Dino = unclassified Dinophyceae; DG-II-10+11 = Syndinophyceae Group II clade 10 and 11; DG-II-6 = Syndinophyceae Group II clade 6; DG-I-1 = Syndinophyceae Group I clade 1; DG-I-4 = Syndinophyceae Group I clade 4; DG-I-5 = Syndinophyceae Group I clade 5; R-P = Raphid pennate diatoms; Ostr = Ostreococcus; Bathy = Bathycoccus and Micro = Micromonas. For (**d**) the taxa codes are: OM1-Act = Actinomarina OM1 clade; Pro = *Prochlorococcus*; Syn = *Synechococcus*; Rho-A169 = Rhodospirillaceaea AEGEAN-169 group; Rhod = uncultured Rhodobacteraceae; SAR11-S1 = SAR11 surface 1; SAR86 = SAR86. For all plots the environmental variables are: Ni = Nitrate; Nt = Nitrite; Ph = Phosphate; Si = Silicate; Temp = Temperature and Sa = Salinity. Please note the differences in the scales.

Overall, CCA models (removing the central region due to missing nutrient data) for both eukaryotes and prokaryotes were significant (p < 0.001 and p = 0.01 respectively). Surface samples from the southern fall stations were typified by higher temperatures and the increase in abundance of *Micromonas*, as well as raphid pennate diatoms (Fig. 4a). The Chlorophyte genus *Bathyococcus* was associated with the higher nutrient concentrations observed in the DCM of the southern fall stations while the abundance of the genus *Ostreococcus* responded positively to depth. For the prokaryotic component, the environmental variables measured could not account for the distribution of the most abundant groups (Fig. 4b).

Mantels tests between showed that, with the exception of the prokaryotic unweighted comparison, the communities showed similar patterns to the environmental data (Table 1).

**Table 1:** Mantel test results for the OTU dissimilarity matrices resulting from the different datasets (Eukaryotic and Prokaryotic) with the environmental distance matrix.

	R	p
Eukaryote_weighted	**0.2352**	**0.002**
Eukaryote_unweighted	**0.3217**	**0.001**
Prokaryote_weighted	**0.292**	**0.001**
Prokaryote_unweighted	0.095	0.062

**Figure 5:**
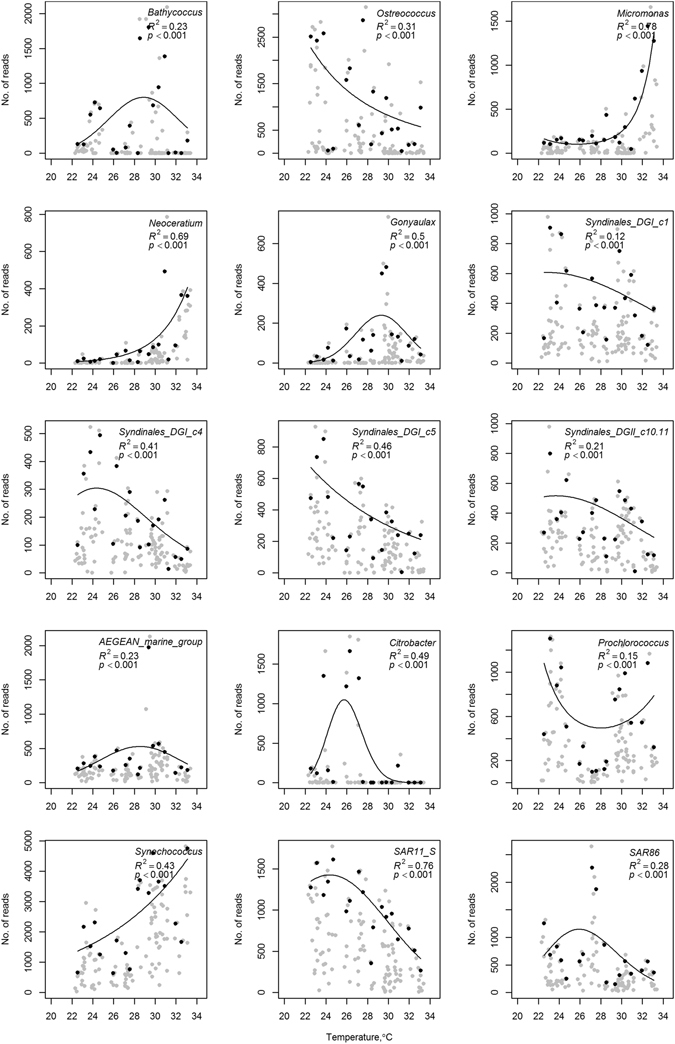
Maximum abundance models for various taxa against temperature. Grey points represent the data while black dots are the 95^th^ percentile of the maximum abundance.

Based on the 95^th^ percentile maximum abundance models, we observed that the Syndiniophyce groups (Group I clades 1, 4 and 5 and Group II clade 10+11), the Mamiellophyceae genus *Ostreococcus* and the Alphaproteobacteria SAR11 responded negatively to the increase in temperature towards the maximum of 34 °C. In contrast, as temperature increased, *Synechococcus* (Cyanobacteria), *Neoceratium* (Alveolata) and *Micromonas* (Mamiellophyceae) became more abundant. *Bathycococcus* (Mamiellophyceae), *Gonyaulax* (Alveolata) and SAR86 (Gammaproteobacteria) had intermediate peaks in abundance while *Prochlorococcus* (Cyanobacteria) showed a bimodal distribution (Fig. 5). What is more, even within the same genus, different responses were observed. For example, the two most abundant OTUs assigned to *Synechococcus* showed different responses to changes in temperature (Supplementary Figure 5).

## Discussion

### Overall distributions

#### Autotrophs

Increased temperatures in nutrient-limited conditions are primarily thought to influence plankton distribution through physical mechanisms (e.g., stratification and nutrient supply) favoring the picoplankton^36^. The dominance of picoplankton has been observed in several oligotrophic regions including the Mediterranean Sea (e.g., refs 37 and 38), the Sargasso Sea^39^, and the Atlantic^40^. In the Red Sea, picoplankton has been shown to account for over 90% of the primary production in ref. 4. The picoplankton comprises cyanobacteria (e.g., genera *Prochlorococcus* and *Synechococcus*), as well as a diverse assortment of small eukaryotic algal taxa. *Synechococcus* is ubiquitous in the marine environment although it is more abundant in more nutrient-rich regions while *Prochlorococcus* is more restricted to oligotrophic tropical and sub-tropical waters^41, 42^. In tropical and sub-tropical regions, High performance liquid chromatography (HPLC) analysis indicated that *Prochlorococcus* could account for a large proportion of the phototrophs (e.g., refs 43–45) and it has previously been shown, using molecular methods, to account for up to 91% of the picocyanobacteria in the Red Sea^13^. Surprisingly, in the current study, except in the northern region in the spring, where *Prochlorococcus* reads numbers equaled those of *Synechococcus*, the latter was the dominant cyanobacterial genus in the Red Sea. The study conducted by Ngugi *et al*.^13^ was conducted in the northern Red Sea and during the spring, attenuating the differences observed between our and their study. Veldhuis & Kraay^46^ found that at the surface, *Synechococcus* abundance was higher than that of *Prochlorococcus*.

While cyanobacteria were large contributors to the 16 S rRNA gene libraries, and undoubtedly played a substantial role in the primary production, in the eukaryotic fraction, Dinophyceae were the dominant plastid-containing taxa throughout the Red Sea, in agreement with previous findings for this^8, 20^, and other oligotrophic regions (e.g., refs 8, 47 and 48). Being mixotrophic protists, they gain energy from sunlight and acquire inorganic nutrient requirements and essential organic nutrients, such as amino acids and vitamins, via bacterivory^49, 50^. This may be especially important in oligotrophic regions where high light levels are present, which could selectively favor mixotrophic grazers over heterotrophs^51^. The mixotrophic nature of Dinophyceae may allow for the propagation of dinoflagellates in oligotrophic conditions, especially in warmer conditions where grazing is reported at higher rates^52^ but in more nutrient replete regions they are likely to be outcompeted by other phototrophs^53^.

In the central Red Sea, although seasonal differences were unable to be determined with the current dataset, the eukaryotic community in this region was generally distinct from either the north or south with the predominance of prasinophyte clade VII A reads. This clade has previously been observed in mesotrophic regions in the Pacific^54, 55^ as well as in the Red Sea during the Tara Ocean cruise^56^. The dominant clade in the Red Sea during the Tara Oceans project (A4) was proposed to be a more coastal strain and although in the current study it was observed in open water this could be due to influence from the water column mixing, which can result from eddy structures that are present in this area creating the conditions for the upwelling of nutrients into the photic zone. However, inter annual variability or the effect of using different filter types, cannot be ruled out as the reason for the differences between the regions in this study.

### Heterotrophs

The most dominant heterotrophic class in the prokaryotic fraction was Alphaproteobacteria. As is typical of other open ocean environments, the dominant group in this class is SAR11 (e.g., refs 57–59), specifically SAR11 - Surface 1, typical of the photic zone^60^. This group was dominant throughout the sampling irrespective of region, depth and season. The apparent cosmopolitan behavior observed in the present study is probably due to the inability of the 16 S rRNA gene to detect ecological differentiation within the clade that is known to have a high level of diversity in phylotypes^13^. SAR86 of the class Gammaproteobacteria, which has previously been reported in oligotrophic waters in the Pacific^61^, was prevalent in the spring when nutrients are limited but detectable. These groups with their small size and genome streamlining have a selective advantage in nutrient limited regions due to resource specialization^62^. It has been suggested that the specialization on different carbon compounds of SAR11 and SAR86 allows them to limit competition and thus have high abundances in the open ocean^62^.

Syndiniophyceae, parasitic members of the Alveolata are known to account for a large proportion of Alveolata reads in marine systems^63, 64^. The dominant groups in the euphotic zone belong to Group I (clades 1, 4 and 5) and Group II (clades 6, 7 and 10+11)^63^. Syndiniophyceae are likely to be highly opportunistic and infect a variety of hosts including dinoflagellates^65^ and radiolarians^66^. The proportion and diversity of parasites in the Red Sea may have a substantial impact on biogeochemical cycles. As well as releasing dissolved organic material into the environment, due to the destruction of host cells, the production of dinospores provides a nutrition source for crustacean zooplankton. This activity also returns a proportion of the energy to higher trophic levels, which could otherwise be lost due to the sinking of larger phytoplankton cells^67, 68^.

### Seasonal and regional variations in the distributions of taxa

**Figure 6:**
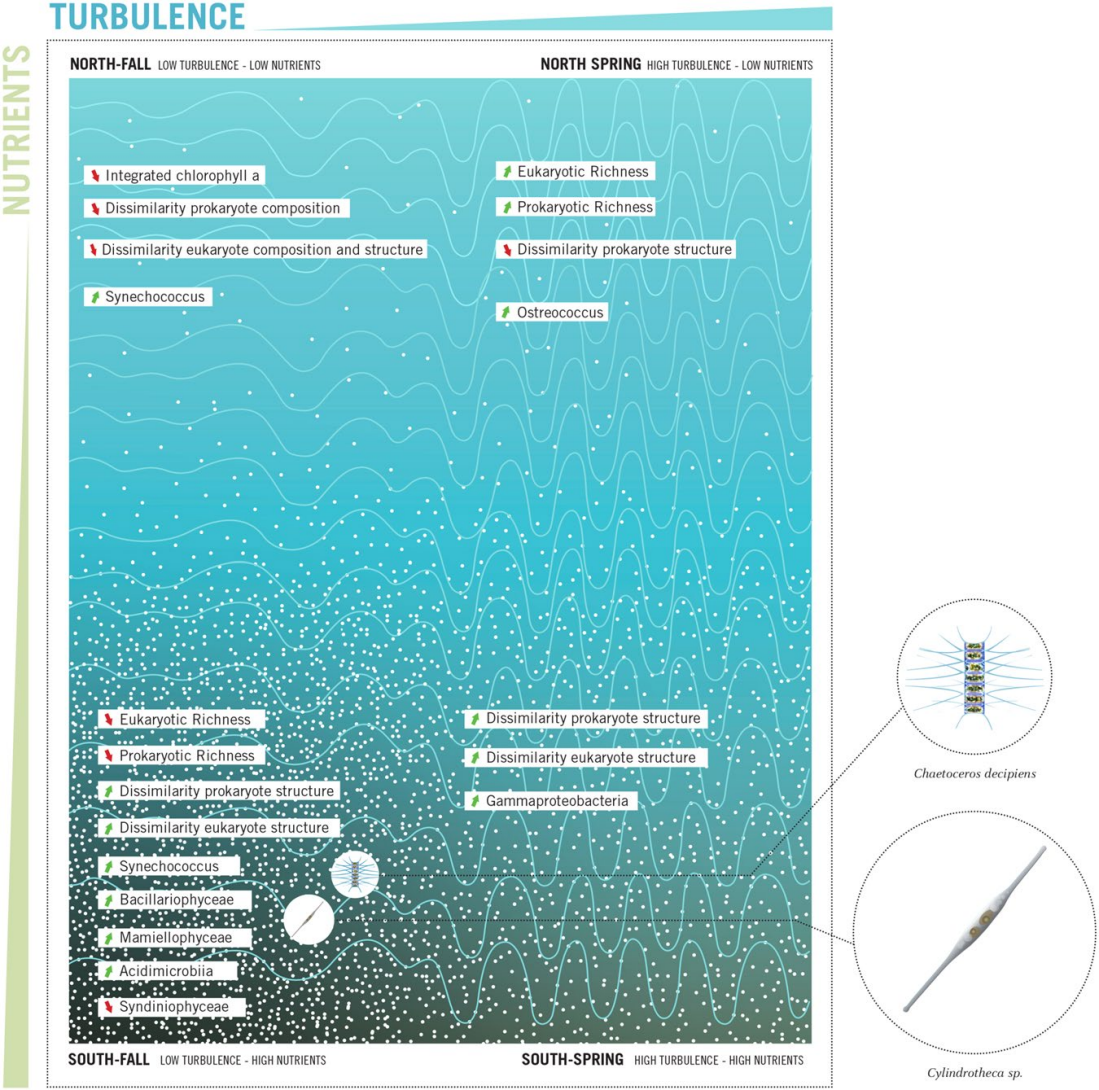
Generalized depiction of the responses of the microbial community in the Red Sea to variations in nutrients and turbulence.

The lowest average richness observed in the current study was observed in the southern region in the fall (Fig. 6), a period of stratification, i.e. low turbulence, and high nutrients input through the GAIW (a relatively cold, nutrient rich and low salinity water mass; 31). With the increase in nutrients, small cells are no longer selected for, as fast growing larger taxa such as diatoms can effectively compete for the available nutrients. Also, in a stratified environment these larger cells may be able to escape grazing pressure as the rates of contact between predator and prey are lower in less turbulent regions^69^. The dominance of diatoms in these conditions deviates slightly from previous concepts^23, 24^ where bloom-forming dinoflagellates are proposed to dominate. However, the N form prevalent in the water column may determine whether bloom forming dinoflagellates or diatoms are present with inorganic forms of N favoring the latter^25^. The low richness observed under a scenario of high nutrient availability is also in agreement with Irigoien *et al*.^70^ who showed a reduction of biodiversity at high levels of phytoplankton biomass. The highest levels of richness were driven by high turbulence and low nutrients. Barton *et al*.^71^ proposed that turbulence can increase the flux of nutrients towards the cell and so increase the cell’s resource affinity. Also, as turbulence can increase the number of predator prey interactions^69^, zooplankton can prevent smaller sizes from consuming all the available resources. Therefore, larger cells may be able to compete with smaller cells in turbulent conditions, reducing competitive exclusion and increasing richness.

The northern Red Sea in the fall is characterized by being highly stratified (low turbulence) and having low nutrient concentrations. In regimes where nutrients and turbulence are both low the microbial loop or specialist groups such as mixotrophs would dominate the food web^24, 25^. Indeed, the current study showed that cyanobacteria dominated the prokaryotic fraction while Dinophyceae, which contain a large proportion of mixotrophic taxa, and the parasitic Syndiniophyceae were the dominant eukaryotic groups. The highest average similarity within a region/season was observed here. This is not surprising, as when perturbations are kept at a low frequency similarity will increase^72^. In general, in the current study, increases in dissimilarity were observed as nutrient concentrations increase especially for the eukaryotes. The relationship between higher nutrients and dissimilarity has been shown previously (e.g., ref. 73) and could be due to the fact that high productivity tends to favor instability and compositional turnover^74^.

### Determination of niches

As sea surface temperatures increase and the size of oligotrophic regions expand^75, 76^, taxa will respond to the changes in the environmental conditions depending on their genomic characteristics^77^. Understanding how taxa will respond to these climatic changes is important to understand how biogeochemical cycles will be affected.

Raising temperatures are predicted to favor small groups, such as cyanobacteria over large phytoplankton (e.g., diatoms)^78^. Our study suggested that, as temperatures increase toward 34 °C, the abundance of reads of *Synechococcus* increased while *Prochlorococcus* showed a bimodal distribution with peaks around 24 and 30 °C. The bimodal distribution of *Prochlorococcus* suggests strain-level differences in their distribution. Low light and high light clades have been previously described for *Prochlorococcus* ^79, 80^. Further, and specifically in the Red Sea, Shibl *et al*.^81, 82^ showed differences in the composition of *Prochlorococcus* in the water column, with strains adapted to conditions lower in the water column possibly having a different thermal tolerance. The peak in abundances around 30 °C is in agreement with culture tests on high light II cultures of *Prochlorococcus* whose growth rate declines rapidly around 28–29 °C^83^. The increased abundances of *Synechococcus* in warmer temperatures is also in agreement with Moore *et al*.^84^ who showed that the growth optimum of *Synechococcus* is higher than that of *Prochlorococcus*. With higher growth optimum, it is possible that *Synechococcus* are better adapted to take advantage of the nutrient inputs into the southern Red Sea (which is also warmer) in the summer and so increase their abundances relative to *Prochlorococcus*. This is especially true as *Synechococcus* is able to take advantage of the influxes of nitrate, such as those from the GAIW, whereas most strains of *Prochlorococcus* seem unable to^80, 85^. Temperature related growth optima are likely to be clade-specific^84^, which is further supported by the current findings.

Three of the most important eukaryotic players in the photic zone of Red Sea waters^14^, the genera of picoeukaryotic green algae (*Bathyococcus*, *Micromonas* and *Ostreococcus*; class Mamiellophyceae), showed distinct responses to depth and temperature. *Bathycoccus* was observed predominantly at the DCM, which seems to be typical of its distribution in other oceans^86, 87^ and is likely to be linked to the increased availability of nutrients at this depth^86^. In contrast, *Micromonas* has a higher affinity to surface waters, particularly near the coast, in line with reports in other biogeographical studies^16, 64, 88, 89^. Meanwhile, *Ostreococcus* showed a more even distribution in the photic zone of the water column. Its abundance was in general higher in the DCM than at the surface^86, 90^. Increased abundances of *Ostreococcus* were recorded in the colder waters of the northern Red Sea during the spring although this may be associated with an increase in the amount of nutrients available in the photic zone due to convective mixing of the water column, which has previously been shown to support high levels of phytoplankton until stratification occurs^91^.

Limited knowledge is available concerning the distribution and habitat preferences of the Syndiniophyceae. While it seemed that the major clades identified in the current study were generally observed throughout the water column, a preference toward lower surface seawater temperature was apparent. While temperature may play a role in the distribution of these parasitic clades, other studies have suggested that the abundance of the host^92^ and nutrient availability^65^ were driving factors in the abundance patterns of for example *Amoebophyra* spp. Further work will have to be undertaken to determine the host species of this diverse collection of parasites to fully understand how the distribution of these groups alters throughout the Red Sea, and in general how they respond to changes in temperature or depth. A greater understanding of the role of parasites in the marine system is required as they play an important role in energy transfer in the marine environment and this is especially important in oligotrophic regions where recycling of organic matter is vital. They may play a role in the control of harmful algal blooms^92^, which are becoming more prevalent^93^.

The prevalence of SAR11, a typical oligotrophic group in Atlantic waters, and a substantial contributor to the Red Sea planktonic community, has been proposed to increase its prevalence as oceans become warmer^94^ and for some strains more oligotrophic^95^. Field data from the warm Red Sea do not fully support this theory as maximum abundance modeling suggests that at the higher temperatures observed in the Red Sea they are not at their growth optima and abundances decline. The warmer water conditions in the Red Sea were also associated with increased nutrients and consequently more mesotrophic groups are likely to be able to take advantage of the nutrient conditions and to selectively out-compete SAR11.

## Conclusion

The present study reinforces the relevance of the Red Sea as a natural laboratory for a better understanding of the responses of the plankton to changes in the physio-chemical environment. By looking at different regions and seasons, we were able to identify different scenarios aligned with the categories proposed by Cullen *et al*.^24^, testing their theory based on field data. Current results show that increasing nutrient levels tended to increase community dissimilarity within a region, especially for the eukaryotes, while higher turbulence, indicated by a deeper mixed layer, was associated with higher OTU richness. Phytoplankton communities in the Red Sea responded similarly to the framework proposed by Cullen *et al*.^24^, with the mixotrophic eukaryotes and cyanobacteria predominant in low turbulence and nutrient conditions being replaced by mesotrophic groups (Bacillariophyceae and Mamiellophyceae) as nutrient levels increased. Picophytoplankton (e.g. *Ostreococcus*), which are specialized to efficiently utilize light and nutrients, dominated the high turbulence low nutrient regimes as proposed by Cullen *et al*.^24^. The use of molecular techniques also allowed a broader assessment of the planktonic community to be assessed compared with Cullen *et al*.^24^ who focused predominantly on phytoplankton.

The present study shows different distributional patterns for the three main groups of Mamiellophyceae (*Bathycoccus*, *Ostreococcus* and *Micromonas*), which were dominant in various parts of the Red Sea. Based on this field-based dataset and on molecular data, we were also able to identify increases in *Synechococcus*, the photosynthetic cyanobacteria, with temperature although this response was shown to be specific to different OTUs. Further investigations of the distributional patterns of taxa and their relationships with other groups will give a more comprehensive understanding of the trophic structure in oligotrophic regions where mixotrophy and parasiticism are likely to be important nutritional modes.

## Methods

### Sample collection

**Figure 7:**
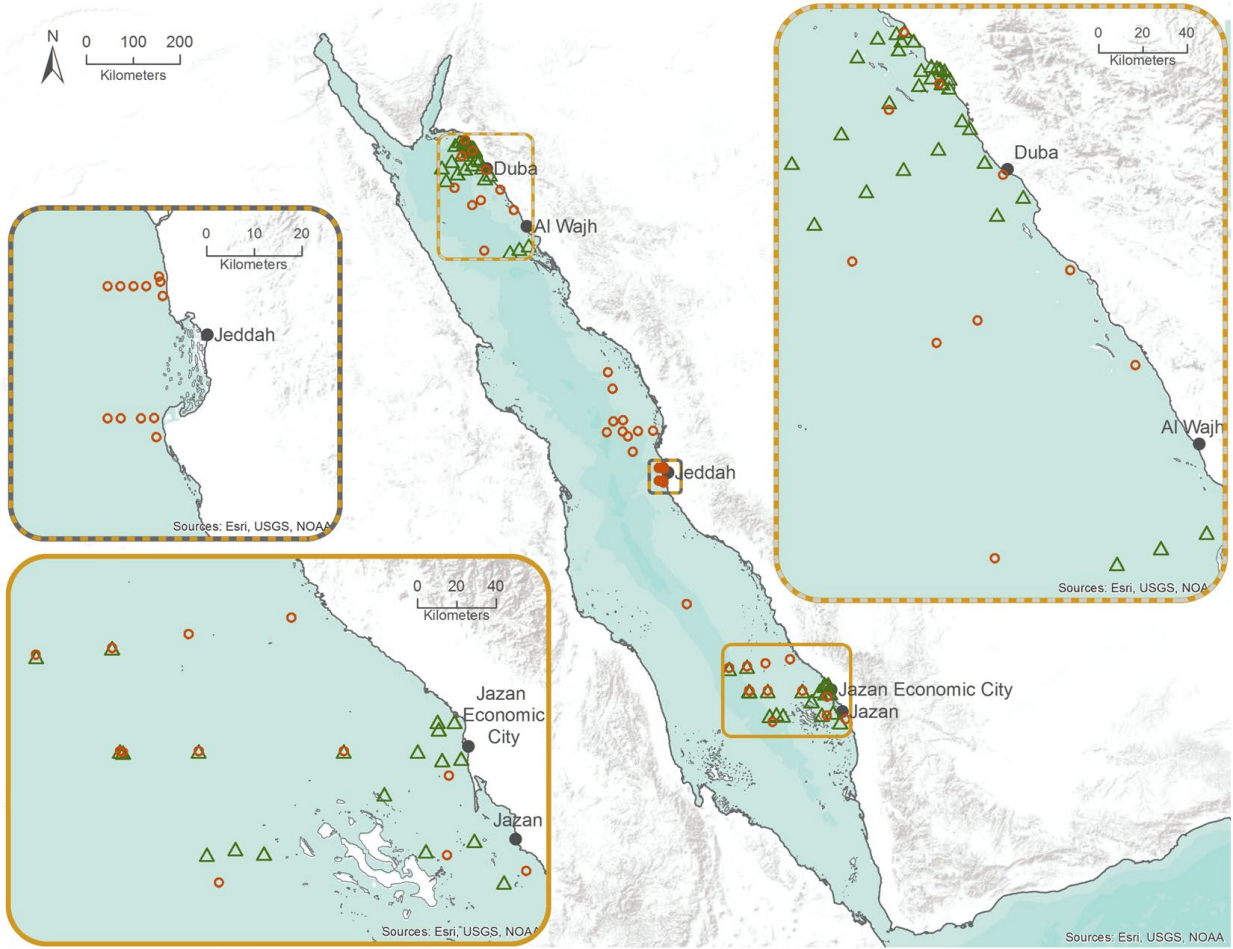
Sampling stations in the north, central and south of the Red Sea. Green triangles represent fall sampling points while orange circles are spring sampling. The map was made in ArcGIS (version: 10.3.1; https://www.arcgis.com/).

Samples were collected during four research cruises undertaken in August and September 2014 (as detailed in 16) and February and April 2015 at both the surface (5 m) and the deep chlorophyll maximum (DCM). A total of 33 stations were sampled in the north of the Red Sea in August and 10 in February while 20 and 13 were sampled in the south of the Red Sea in September and April respectively (Fig. 7). A fifth cruise was undertaken during March/April 2013 in the central region of the Red Sea (21 stations). The coordinates of the stations for each sampling point are presented in Supplementary Table 1 (alongside ancillary information such as sampling depth, temperature, salinity, chlorophyll *a*, nitrate, nitrite, phosphate and silicate concentrations, mix layer depth).

Sampling was undertaken at 5 m (described as the surface) and the DCM, with the DCM being determined during the downcast of the CTD/rosette profiler. During the upcast, 10 L Niskin bottles were triggered for closure at the desired depth.

From the 10 L Niskin bottles, 5 L of water (with no prefiltration) was filtered through either 0.22 µm membrane filters (Millipore) (used during the cruises to the north and south of the Red Sea) or through a 0.22 µm CellTrap (MemTeq) during the cruise in the central region. Individual filters were stored in 15 mL tubes with ~5 mL lysis buffer and frozen at −20 °C until analysis. The concentrated cell samples from the CellTrap were immediately frozen at −20 °C.

Water, for nutrient analysis and chlorophyll *a*, was collected at the same depths, which were sampled for DNA analysis. The method for nutrient analysis and chlorophyll *a* is as described in Pearman *et al*.^16^.

The mixed layer depth was ascertained from density measurements calculated from temperature and salinity values obtained from the rosette profiler. The mixed layer depth was used as a proxy for turbulence (e.g. A deep mixed layer equates to high turbulence whilst a small mixed layer equals a stratified and stable water column).

### DNA extraction and PCR amplification

Concentrated cells from the CellTraps were centrifuged at 21 000 × *g* for 30 min prior to being re-suspended in 180 μL ATL buffer (Qiagen) and 20 μL proteinase K (20 mg mL^−1^). The filters were removed from the falcon tubes, placed in 2 mL eppendorfs with 540 μL ATL buffer (Qiagen) and 60 μL proteinase K (20 mg mL^−1^) added to them. Sample tubes from both methods were incubated at 55 °C for 30 min and DNA extraction and PCR amplification followed the same procedure as described in Pearman *et al*.^16^. The primers used for amplification targeted the v3 and v4 regions of the 16S rRNA gene^96^ and the v4 region of the 18S rRNA gene^97^. Subsequent to the PCR amplification (where a no negative control was also run) samples were cleaned and normalized using a SeqPrep Normalization plate prior to MiSeq library preparation. The library was prepared following the Illumina 16S metagenomic sequencing library preparation protocol. Before sequencing the samples were cleaned and normalized a second time and tagged samples were pooled for sequencing on a MiSeq sequencing platform at the King Abdullah University Core Laboratory. Raw reads were submitted to the NCBI SRA archive and can be accessed under the project accessions: SRP060785 and SRP081162.

### Data analysis

Automatic demultiplexing of samples occurred during the MiSeq sequencing. The forward and reverse raw reads were joined using the join_seqs.py script in QIIME^98^ and quality filtered (phred = 25). Quality filtered reads were concatenated and the forward and reverse primers removed in mothur^99^. Any reads not containing the forward or reverse primers (with no errors) were removed from the analysis. Clustering of reads in OTUs followed a two-step process in QIIME. Firstly CD-HIT^100^ was implemented with the trie function before representative sequences were selected (first sequence of cluster was selected). A second round of clustering was undertaken using USEARCH (version 5.2.236)^101^ at 97% similarity with a minimum cluster size of two. During the USEARCH clustering chimeras were detected using both a denovo approach and against a reference database (SILVA 123^102^). Reference sequences obtained from the USEARCH clustering were taxonomically identified using the PR2^103^ database for eukaryotes and the SILVA 123 database for prokaryotes using the uclust algorithm. Sequences not assigned to at least the phylum level were removed from the analysis as were sequences relating to Metazoa in the eukaryotic fraction (due to these organisms not being representatively sampled using Niskin bottles) and chloroplasts (due to being from eukaryotic organisms) from the prokaryotic dataset.

To ensure an even depth of sequencing per sample reads were rarefied to 5000 reads per sample for the eukaryotes and 5700 reads for the prokaryotes multiple times (n = 100). Samples not reaching this threshold were subsequently removed from the analysis. The composition of the taxonomic groups was assessed using the R^104^ package *phyloseq* ^105^ using the average number of reads per region/depth/season and visualized with *ggplot* ^106^. Faith’s phylogenetic distance (PD) measure of alpha diversity was calculated in QIIME and analysis of variance (ANOVA) statistics were calculated in R. To achieve this the data was subset into a regional subset and a seasonal subset. The regional subset contained all the regions (north, central and south) but only for the spring. A two-way ANOVA for region (three levels: North, Central and South) and depth (two levels: Surface and DCM) was undertaken on this subset. For the analysis of seasons the subset contained data for both seasons (fall and spring) and the regions north and south. A three-way ANOVA was undertaken with the factors region (two levels: North and South), depth (two levels: Surface and DCM) and season (two levels: Spring and Fall). ANOVA was used to test the dissimilarity of the communities (measured with weighted and unweighted Unifrac) with four levels to the factor (North_Fall, North_Spring, South_Fall and South_Spring).

Non-metric multidimensional scaling (NMDS) ordination was undertaken using weighted and unweighted UniFrac^107^ distance matrices within the framework of the package *phyloseq*. Using the PRIMER v6 package^108^ with the PERMANOVA + add on^109^ a three-way PERMANOVA was undertaken. This assessed the significance of the factors “region” (orthogonal three levels, North, Central and South), “season” (orthogonal two levels, fall and spring) and “depth” (orthogonal two levels, surface and DCM). The statistical significance was tested using 9999 permutations of the residuals under a reduced model with a significance level of α = 0.05. Those effects, which were significant, were further investigated through a series of pairwise comparisons. Constrained Correspondence Analysis (CCA) was undertaken on the OTU tables merged at the level of genus. The CCA model was tested for significances using the permutational ANOVA (permutations = 999) within the *vegan* ^110^ package of R. Comparative (Mantel-type) tests were undertaken on the community dissimilarity matrices and an environmental distance matrix (Eucilidean) in *vegan* using the Pearson’s rank method.

Species Distribution Models based on maximum abundances across environmental gradients of depth, temperature and salinity were modeled using the method proposed by Blackburn *et al*.^111^. For these models, each of the three variables (depth, temperature, salinity) was divided into categories. Each category included no more than 20 observations, and there were roughly equal numbers of observations in at least three categories. For each category, the 95^th^ percentile abundance was calculated for each taxon. For all taxa, regressions were conducted using the number of observations in each category as a weighting. Generalized Linear Models (GLMs) were fitted to the data, using the 95^th^ percentile in each category as the dependent variable, a log link function and a Poisson likelihood function. The independent variable offered to the GLM included up to three-degree polynomials. The number of higher degree terms entered into the final model was based on the Akaike Information Criterion (AIC) and only higher degree terms that increased the proportion of deviance explained by more than 1% were included. The final model used for each taxon was that function which explained the most variability. All analyses were conducted using the R software package.

## Electronic supplementary material

Supplementary Information
