# Supplementary material for: Microbial planktonic communities in the Red Sea: high levels of spatial and temporal variability shaped by nutrient availability and turbulence
Source: Sci Rep. 2017 Jul 26;7:6611. doi: 10.1038/s41598-017-06928-z (PMC5529573; doi:10.1038/s41598-017-06928-z)
Supplement: Supplementary file 1 — Supplementary Information [file 41598_2017_6928_MOESM1_ESM.doc]

**Microbial planktonic communities in the Red Sea: high levels of spatial and temporal variability shaped by nutrient availability and turbulence**

John. K. Pearman^1^, Joanne Ellis^1^, Xabier Irigoien^1,2^, Y.V.B Sarma^1^, Burton H. Jones^1^, Susana Carvalho^1^

^1^ King Abdullah University of Science and Technology (KAUST), Red Sea Research Center (RSRC), Biological and Environmental Sciences and Engineering (BESE), Thuwal, 23955-6900, Saudi Arabia

^2^ Current address: AZTI tecnalia, Herrera Kaia, Portualedea z/g Pasaia Gipuzkoa 20110, Spain

Corresponding Author: John K. Pearman. Email: john.pearman@kaust.edu.sa

Supplementary Figure 1: Heat map with prokaryotes in the bottom triangle and eukaryotes in the top triangle.

Supplementary Figure 2: Concentrations of nutrients (Nitrate, Phosphate, Nitrite and Silicate) and dissimilarity results for weighted and unweighted UniFrac for prokaryotes and eukaryotes. Letters denote those categories, which are similar based on the ANOVA results in Supplementary Table 2.

Supplementary Figure 3: The proportion of reads (with standard error bars) in the different regions, depths, and seasons for the three most dominant Mamiellophyceae genera present throughout the Red Sea.

Supplementary Figure 4: The proportion of prokaryotic reads (with standard error bars) in the different regions (north, south), depths (surface, deep chlorophyll maximum – DCM), and seasons (spring, fall) for the two most dominant Cyanobacteria genera present throughout the Red Sea.

Supplementary Figure 5: Response of the two most abundant *Synechococcus* OTUs (a) Syn 2475 and b) Syn1690 to temperature.

Supplementary Table 1: Ancillary information of sampling points including latitudinal and longitudinal coordinates, sampling depth, chlorophyll a concentration, nutrient concentrations (Nitrate, Nitrite, Phosphate and Silicate), Temperature, Salinity, Mix layer depth and integrated chlorophyll and Faith’s diversity for prokaryotes and eukaryotes.

| Sample ID | Latitude | Longitude | Depth | Chlorophyll a | Nitrate (uM) | Nitrite (uM) | Phosphate (uM) | Silicate (uM) | Temperature | Salinity | Mix layer depth (m) | Integrated chlorophyll a (mg m-3) | PD Prokaryotes | PD Eukaryotes |
| --- | --- | --- | --- | --- | --- | --- | --- | --- | --- | --- | --- | --- | --- | --- |
| E11D.spring | 22.310 | 38.890 | 70.010 | NA | NA | NA | NA | NA | 25.588 | 39.467 | NA | NA | NA | 30.215 |
| E11S.spring | 22.310 | 38.890 | 5.020 | NA | NA | NA | NA | NA | 26.959 | 38.938 | NA | NA | 19.148 | 25.952 |
| E1D.spring | 23.437 | 38.020 | 50.230 | NA | NA | NA | NA | NA | 25.133 | 39.616 | NA | NA | 20.622 | 27.798 |
| E1S.spring | 23.437 | 38.020 | 6.900 | NA | NA | NA | NA | NA | 26.108 | 39.322 | NA | NA | 28.611 | 23.927 |
| E2S.spring | 23.120 | 38.110 | 6.740 | NA | NA | NA | NA | NA | 26.540 | 38.889 | NA | NA | 24.734 | 28.138 |
| E4S.spring | 22.510 | 38.310 | 5.850 | NA | NA | NA | NA | NA | 26.348 | 39.012 | NA | NA | 30.429 | 18.313 |
| E5D.spring | 22.200 | 38.400 | 79.550 | NA | NA | NA | NA | NA | 25.089 | 39.626 | NA | NA | 23.851 | 18.218 |
| E5S.spring | 22.200 | 38.400 | 4.970 | NA | NA | NA | NA | NA | 26.602 | 39.046 | NA | NA | NA | 17.018 |
| E6S.spring | 21.900 | 38.500 | 5.100 | NA | NA | NA | NA | NA | 26.929 | 38.913 | NA | NA | 13.907 | 10.360 |
| E8D.spring | 22.280 | 38.000 | 71.150 | NA | NA | NA | NA | NA | 25.557 | 39.498 | NA | NA | NA | 24.291 |
| E10S.spring | 22.300 | 38.600 | 7.880 | NA | NA | NA | NA | NA | 26.646 | 39.000 | NA | NA | 24.554 | 5.153 |
| E9D.spring | 22.290 | 38.300 | 59.600 | NA | NA | NA | NA | NA | 25.842 | 39.342 | NA | NA | NA | 3.604 |
| E9S.spring | 22.290 | 38.300 | 5.080 | NA | NA | NA | NA | NA | 27.009 | 39.035 | NA | NA | 22.327 | 25.371 |
| D12D.spring | 27.337 | 35.669 | 40.000 | NA | 2.080 | 0.060 | 0.100 | 1.190 | 22.540 | 40.430 | 35.000 | 0.189 | 41.995 | 32.989 |
| D12S.spring | 27.337 | 35.669 | 6.000 | NA | 0.240 | 0.050 | 0.100 | 0.750 | 23.500 | 40.420 | 35.000 | 0.189 | 34.645 | 21.421 |
| D13D.spring | 26.656 | 35.399 | 65.000 | NA | 0.580 | 0.050 | 0.100 | 1.030 | 23.190 | 40.340 | 71.000 | 0.109 | 34.773 | 50.150 |
| D13S.spring | 26.656 | 35.399 | 5.000 | NA | 0.230 | 0.050 | 0.100 | 0.940 | 24.160 | 40.190 | 71.000 | 0.109 | 36.650 | 28.949 |
| D16D.spring | 26.950 | 35.942 | 70.000 | NA | 1.240 | 0.110 | 0.100 | 1.120 | 22.980 | 40.370 | 120.000 | 0.189 | 32.748 | 33.204 |
| D16S.spring | 26.950 | 35.942 | 5.000 | NA | 0.200 | 0.050 | 0.100 | 0.810 | 23.940 | 40.380 | 120.000 | 0.189 | 24.007 | 13.275 |
| D20D.spring | 26.565 | 36.206 | 80.000 | NA | 1.120 | 0.090 | 0.100 | 1.060 | 22.930 | 40.370 | 118.000 | 0.329 | 26.777 | 25.773 |
| D20S.spring | 26.565 | 36.206 | 5.000 | NA | 0.200 | 0.050 | 0.100 | 0.940 | 23.930 | 40.360 | 118.000 | 0.329 | 23.874 | 30.077 |
| D21D.spring | 25.782 | 35.634 | 98.000 | NA | 1.960 | 0.130 | 0.100 | 0.930 | 23.390 | 40.330 | 112.000 | 0.320 | 41.209 | 47.639 |
| D21S.spring | 25.782 | 35.634 | 5.000 | NA | 0.570 | 0.030 | 0.100 | 0.950 | 24.070 | 40.180 | 112.000 | 0.320 | 38.363 | 38.924 |
| D4D.spring | 27.916 | 35.266 | 50.000 | NA | 0.740 | 0.050 | 0.100 | 1.010 | 23.020 | 40.490 | 109.000 | 0.292 | 33.655 | 45.135 |
| D4S.spring | 27.916 | 35.266 | 5.000 | NA | 0.080 | 0.050 | 0.100 | 0.890 | 23.120 | 40.410 | 109.000 | 0.292 | 40.972 | 47.331 |
| D5D.spring | 26.747 | 35.565 | NA | NA | 5.200 | 0.170 | 0.190 | 1.830 | 22.320 | 40.520 | NA | NA | 41.626 | 36.568 |
| D5S.spring | 26.747 | 35.565 | 6.000 | NA | 0.340 | 0.050 | 0.100 | 0.520 | 22.680 | 40.470 | NA | NA | 26.273 | 40.641 |
| D7D.spring | 27.601 | 35.206 | 70.000 | NA | 0.770 | 0.160 | 0.100 | 0.950 | 22.550 | 40.430 | 106.000 | 0.269 | NA | 35.399 |
| D7S.spring | 27.601 | 35.206 | 5.000 | NA | 0.080 | 0.050 | 0.100 | 0.930 | 23.220 | 40.420 | 106.000 | 0.269 | 38.291 | 37.031 |
| D8D.spring | 27.708 | 35.410 | 45.000 | NA | 2.410 | 0.100 | 0.100 | 1.320 | 22.700 | 40.420 | 78.000 | 0.105 | 40.502 | 32.341 |
| D8S.spring | 27.708 | 35.410 | 5.000 | NA | 0.220 | 0.050 | 0.100 | 0.920 | 23.120 | 40.430 | 78.000 | 0.105 | 34.977 | 27.638 |
| D9D.spring | 26.986 | 35.057 | 80.000 | NA | 0.100 | 0.050 | 0.100 | 0.410 | 22.450 | 40.450 | 76.000 | 0.308 | 40.187 | 44.253 |
| D9S.spring | 26.986 | 35.057 | 5.000 | NA | 0.090 | 0.050 | 0.100 | 0.900 | 23.210 | 40.350 | 76.000 | 0.308 | 31.515 | 41.750 |
| DI1S.fall | 27.527 | 35.534 | 5.000 | 0.351 | 0.519 | 0.062 | 0.100 | 0.342 | 30.080 | 40.136 | NA | NA | 33.622 | 42.751 |
| DS10D.fall | 27.250 | 35.751 | 73.400 | 0.233 | 0.638 | 0.061 | 0.100 | 0.958 | 24.169 | 40.145 | 11.000 | 0.070 | 40.817 | 52.817 |
| DS10S.fall | 27.250 | 35.751 | 5.000 | 0.135 | 0.456 | 0.050 | 0.100 | 0.748 | 29.639 | 40.203 | 11.000 | 0.070 | 30.617 | 44.683 |
| DS12D.fall | 27.390 | 35.594 | 62.580 | 0.278 | 0.421 | 0.063 | 0.100 | 0.998 | 24.630 | 40.089 | 8.000 | 0.059 | 39.107 | 45.205 |
| DS12S.fall | 27.390 | 35.594 | 6.000 | 0.105 | 0.435 | 0.050 | 0.100 | 0.698 | 30.400 | 40.215 | 8.000 | 0.059 | 36.370 | 47.981 |
| DS13AD.fall | 27.561 | 35.502 | 59.640 | 0.405 | 0.650 | 0.047 | 0.100 | 0.726 | 24.270 | 40.118 | 10.000 | 0.058 | 40.539 | 54.626 |
| DS13AS.fall | 27.561 | 35.502 | 5.000 | 0.057 | 0.439 | 0.050 | 0.100 | 0.613 | 31.170 | 38.006 | 10.000 | 0.058 | 29.915 | 47.800 |
| DS13D.fall | 27.561 | 35.502 | 56.110 | 0.340 | 0.402 | 0.050 | 0.100 | 0.984 | 24.630 | 40.103 | 7.000 | 0.066 | 31.166 | 45.875 |
| DS13S.fall | 27.561 | 35.502 | 5.000 | 0.057 | 0.463 | 0.050 | 0.100 | 0.508 | 31.170 | 38.006 | 7.000 | 0.066 | 31.701 | 42.476 |
| DS14D.fall | 27.710 | 35.419 | 65.960 | 0.354 | 0.615 | 0.108 | 0.100 | 0.960 | 24.280 | 40.101 | 10.000 | 0.084 | 35.466 | 47.879 |
| DS14S.fall | 27.710 | 35.419 | 5.000 | 0.103 | 0.407 | 0.050 | 0.100 | 0.597 | 30.760 | 40.199 | 10.000 | 0.084 | 30.598 | 49.996 |
| DS15D.fall | 27.734 | 35.378 | 64.060 | 0.284 | 0.855 | 0.098 | 0.100 | 0.957 | 24.290 | 40.097 | 9.000 | 0.073 | 26.848 | 41.573 |
| DS15S.fall | 27.734 | 35.378 | 7.000 | 0.022 | 0.572 | 0.050 | 0.100 | 0.536 | 30.690 | 40.085 | 9.000 | 0.073 | 34.899 | 55.425 |
| DS16S.fall | 27.763 | 35.337 | 5.000 | 0.062 | 0.370 | 0.050 | 0.100 | 0.554 | 29.513 | 39.808 | 9.000 | NA | 20.777 | 42.120 |
| DS17S.fall | 27.848 | 35.246 | NA | 0.089 | 0.446 | 0.050 | 0.100 | 0.570 | NA | NA | NA | NA | 21.907 | 43.407 |
| DS28D.fall | 27.913 | 35.236 | 27.917 | NA | NA | NA | NA | NA | 40.257 | NA | 10.000 | NA | 35.059 | 49.123 |
| DS18S.fall | 27.848 | 35.246 | 5.000 | 0.310 | 0.434 | 0.050 | 0.100 | 0.617 | 28.400 | 39.900 | NA | NA | 22.217 | 34.232 |
| DS28S.fall | 27.913 | 35.236 | 5.000 | 0.152 | 0.434 | 0.050 | 0.100 | 0.617 | 28.420 | 39.891 | 10.000 | NA | 23.914 | 40.928 |
| DS19D.fall | 27.921 | 35.269 | 63.340 | 0.464 | 1.128 | 0.137 | 0.100 | 1.070 | 24.920 | 40.098 | 7.000 | 0.249 | 40.523 | 52.398 |
| D19S.fall | 27.921 | 35.269 | 5.000 | 0.240 | 1.110 | 0.145 | 0.100 | 1.059 | 30.800 | 40.100 | 7.000 | 0.249 | 28.834 | NA |
| DS20S.fall | 27.882 | 35.258 | 6.000 | 0.142 | 0.912 | 0.117 | 0.100 | 0.589 | 30.550 | 40.298 | 9.000 | 0.171 | 24.317 | 49.182 |
| DS21D.fall | 27.882 | 35.306 | NA | 0.310 | 0.495 | 0.050 | 0.100 | 0.896 | NA | NA | 7.000 | 0.143 | 29.127 | 45.951 |
| DS21S.fall | 27.882 | 35.306 | 5.000 | 0.154 | 0.427 | 0.050 | 0.100 | 0.563 | 30.622 | 40.463 | 7.000 | 0.143 | 29.914 | 37.637 |
| DS22S.fall | 27.780 | 35.377 | 5.000 | 0.147 | 0.521 | 0.050 | 0.100 | 0.444 | 29.705 | 39.911 | 8.000 | NA | 18.328 | 39.903 |
| DS23S.fall | 27.709 | 35.426 | 5.000 | 0.060 | 0.470 | 0.050 | 0.100 | 0.593 | 29.809 | 40.229 | 11.000 | NA | 27.369 | 47.080 |
| DS24S.fall | 27.770 | 35.413 | NA | 0.401 | 0.447 | 0.050 | 0.100 | 0.647 | NA | NA | NA | NA | 27.240 | 39.266 |
| DS25S.fall | 27.762 | 35.431 | 5.000 | 0.108 | 0.340 | 0.050 | 0.100 | 0.630 | 29.705 | 39.974 | 7.000 | NA | 29.327 | 46.265 |
| DS26S.fall | 27.728 | 35.452 | 5.000 | 0.054 | 0.577 | 0.050 | 0.100 | 0.585 | 30.012 | 40.179 | 9.000 | NA | 30.756 | 45.881 |
| DS27S.fall | 27.692 | 35.449 | 5.000 | 0.072 | 0.508 | 0.050 | 0.100 | 0.624 | 29.905 | 40.210 | 10.000 | NA | 32.883 | 47.632 |
| DS9S.fall | 27.176 | 35.644 | 5.000 | 0.060 | 0.534 | 0.050 | 0.100 | 0.646 | 30.200 | 40.100 | 7.000 | 0.063 | 15.220 | 47.083 |
| DT10S.fall | 27.775 | 35.400 | 5.000 | 0.080 | 0.456 | 0.050 | 0.100 | 0.748 | 29.600 | 40.200 | NA | NA | 19.584 | 35.317 |
| DT12D.fall | 27.895 | 35.160 | 32.860 | 0.176 | 0.501 | 0.050 | 0.100 | 0.792 | 26.420 | 40.055 | 8.000 | 0.117 | 34.974 | 48.789 |
| DT12S.fall | 27.895 | 35.160 | 6.000 | 0.171 | 0.501 | 0.050 | 0.100 | 0.724 | 30.190 | 40.055 | 8.000 | 0.117 | 28.438 | 44.740 |
| DT13D.fall | 27.818 | 35.077 | 56.100 | 0.850 | 0.402 | 0.050 | 0.100 | 0.984 | 24.600 | 40.100 | NA | NA | 34.367 | 53.510 |
| DT13S.fall | 27.818 | 35.077 | 7.000 | 0.055 | 0.690 | 0.050 | 0.100 | 0.598 | 29.660 | 40.163 | NA | NA | 31.911 | 50.684 |
| DT2D.fall | 27.444 | 35.407 | 74.890 | 0.600 | 0.721 | 0.052 | 0.100 | 1.002 | 23.790 | 40.132 | 14.000 | 0.029 | 35.425 | 41.074 |
| DT2S.fall | 27.444 | 35.407 | 6.000 | 0.025 | 0.487 | 0.050 | 0.100 | 0.428 | 30.910 | 40.006 | 14.000 | 0.029 | 32.821 | 37.261 |
| DT3D.fall | 27.362 | 35.265 | 68.010 | 0.310 | 0.707 | 0.059 | 0.100 | 0.978 | 23.680 | 40.143 | NA | NA | 38.354 | 48.985 |
| DT3S.fall | 27.362 | 35.265 | 12.000 | 0.310 | 0.447 | 0.050 | 0.100 | 0.487 | 30.460 | 39.981 | NA | NA | 35.559 | 41.299 |
| DT4D.fall | 27.271 | 35.114 | 100.050 | 0.217 | 0.574 | 0.099 | 0.100 | 1.021 | 23.050 | 40.240 | 28.000 | 0.032 | 36.833 | 52.353 |
| DT4S.fall | 27.271 | 35.114 | 7.000 | 0.038 | 0.471 | 0.050 | 0.100 | 0.415 | 30.480 | 40.053 | 28.000 | 0.032 | 34.815 | 39.774 |
| DT5D.fall | 27.140 | 34.902 | 93.300 | 0.148 | 0.587 | 0.138 | 0.100 | 0.923 | 22.900 | 40.300 | 18.000 | 0.023 | 37.748 | 28.915 |
| DT5S.fall | 27.140 | 34.902 | 5.000 | 0.060 | 0.532 | 0.050 | 0.100 | 0.517 | 30.360 | 40.096 | 18.000 | 0.023 | 34.978 | 40.710 |
| DT6D.fall | 27.386 | 34.811 | 70.860 | 0.372 | 0.776 | 0.061 | 0.100 | 1.045 | 23.190 | 40.195 | 8.000 | 0.027 | 35.137 | 50.217 |
| DT6S.fall | 27.386 | 34.811 | 5.000 | 0.020 | 0.413 | 0.050 | 0.100 | 0.469 | 30.300 | 40.100 | 8.000 | 0.027 | 29.204 | NA |
| DT7D.fall | 27.507 | 35.012 | 78.930 | 0.452 | 0.794 | 0.050 | 0.065 | 1.099 | 23.360 | 40.177 | 22.000 | 0.028 | 24.517 | 33.726 |
| DT7S.fall | 27.507 | 35.012 | 5.000 | 0.056 | 0.545 | 0.050 | 0.100 | 0.382 | 30.150 | 39.936 | 22.000 | 0.028 | 34.682 | 40.839 |
| DT8D.fall | 27.633 | 35.207 | 75.580 | 0.173 | 0.585 | 0.050 | 0.100 | 0.917 | 23.820 | 40.130 | 15.000 | 0.043 | 35.653 | 51.719 |
| DT8S.fall | 27.633 | 35.207 | 6.000 | 0.069 | 0.434 | 0.050 | 0.100 | 0.579 | 30.040 | 40.159 | 15.000 | 0.043 | 31.738 | 46.873 |
| DT9S.fall | 27.703 | 35.327 | 5.000 | 0.091 | 0.495 | 0.050 | 0.100 | 0.561 | 30.670 | 40.138 | 7.000 | 0.083 | 24.299 | 44.621 |
| FI11D.fall | 16.698 | 42.498 | 27.100 | 0.908 | 1.955 | 1.112 | 0.219 | 3.469 | 31.600 | 38.500 | 27.000 | 0.658 | 48.421 | NA |
| FI11S.fall | 16.698 | 42.498 | 2.458 | 0.876 | 0.557 | 0.085 | 0.100 | 1.302 | 33.399 | 38.833 | 27.000 | 0.658 | 30.253 | 37.622 |
| FI14D.fall | 17.102 | 41.951 | 26.100 | 2.540 | 1.796 | 0.335 | 0.273 | 2.500 | 30.300 | 38.700 | 7.000 | 0.943 | 50.352 | NA |
| FI14S.fall | 17.102 | 41.951 | 2.800 | 0.287 | NA | NA | NA | NA | 32.900 | 38.800 | 7.000 | 0.943 | 27.517 | 36.561 |
| FI15D.fall | 16.825 | 41.141 | 34.401 | 0.443 | NA | NA | NA | NA | 27.833 | 37.612 | 8.000 | 0.131 | 34.931 | 38.496 |
| FI15S-2.fall | 16.825 | 41.141 | 2.691 | 0.280 | 0.670 | 0.050 | 0.100 | 0.462 | 32.327 | 38.540 | 8.000 | 0.131 | 43.148 | 39.042 |
| FI15S.fall | 16.825 | 41.141 | 2.691 | 0.280 | 0.670 | 0.050 | 0.100 | 0.462 | 32.327 | 38.540 | 8.000 | 0.131 | 31.718 | 23.488 |
| FI1D.fall | 16.888 | 42.362 | 31.239 | 0.859 | 5.111 | 0.601 | 0.545 | 5.288 | 31.144 | 39.628 | 14.000 | 0.557 | 34.761 | 31.676 |
| FI1S.fall | 16.888 | 42.362 | 31.239 | 0.502 | 0.308 | 0.050 | 0.100 | 1.111 | 31.144 | 39.628 | 14.000 | 0.557 | 26.880 | 36.910 |
| FI5D.fall | 16.840 | 42.142 | 46.058 | 1.018 | 2.441 | 0.718 | 0.100 | 2.364 | 29.723 | 38.521 | NA | 0.828 | 34.906 | 47.430 |
| FI8D.fall | 16.850 | 41.270 | 30.462 | 1.146 | 6.959 | 1.294 | 0.100 | 3.686 | 27.838 | 37.392 | NA | 0.537 | 42.373 | 35.546 |
| FI8S.fall | 16.850 | 41.270 | 5.025 | 1.146 | 0.542 | 0.085 | 0.100 | 1.337 | 32.361 | 29.014 | NA | 0.537 | 29.897 | 42.249 |
| FI9D.fall | 16.831 | 41.400 | 19.982 | 2.481 | 4.988 | 1.056 | 0.581 | 3.741 | 28.629 | 37.531 | 7.000 | 1.555 | 40.214 | 35.874 |
| FI9S.fall | 16.831 | 41.400 | 19.982 | 0.881 | 0.537 | 0.050 | 0.100 | 0.661 | 28.629 | 37.531 | 7.000 | 1.555 | 23.888 | 35.135 |
| FT10D.spring | 16.821 | 42.237 | 16.000 | 1.773 | 0.296 | 0.099 | 0.157 | 1.047 | 27.600 | 38.800 | 65.000 | 0.590 | 47.024 | 52.475 |
| FT10S.spring | 16.821 | 42.237 | 1.500 | 0.577 | 0.123 | 0.058 | 0.169 | 1.323 | 27.100 | 37.900 | 65.000 | 0.590 | 24.195 | 7.865 |
| FT11S.spring | 16.750 | 42.600 | 1.400 | 0.456 | 0.122 | 0.029 | 0.033 | 1.783 | 27.200 | 37.400 | 64.000 | 0.346 | 44.066 | 42.794 |
| FT12D.spring | 17.907 | 41.527 | 32.000 | 0.936 | 0.179 | 0.197 | 0.184 | 0.917 | 27.600 | 38.500 | 40.000 | 0.577 | 47.475 | 46.646 |
| FT12S.spring | 17.907 | 41.527 | 1.700 | 0.266 | 0.129 | 0.053 | 0.168 | 0.798 | 28.300 | 38.100 | 40.000 | 0.577 | 40.668 | 41.230 |
| FT1D.fall | 17.728 | 40.360 | 55.037 | 0.408 | 0.761 | 0.347 | 0.119 | 1.163 | 30.417 | 38.682 | 14.000 | 0.420 | 38.716 | 42.439 |
| FT1S.fall | 17.728 | 40.360 | 55.000 | 0.950 | 0.761 | 0.347 | 0.119 | 1.163 | 30.400 | 38.700 | 14.000 | 0.420 | 34.850 | NA |
| FT2D.fall | 17.767 | 40.706 | 55.624 | 0.708 | 0.644 | 0.194 | 0.116 | 1.159 | 29.140 | 38.716 | 10.000 | 0.110 | 38.791 | 38.750 |
| FT2S.fall | 17.767 | 40.706 | 3.049 | 0.891 | 0.481 | 0.050 | 0.100 | 0.461 | 31.760 | 36.972 | 10.000 | 0.110 | NA | 42.791 |
| FT2S.spring | 17.767 | 40.706 | 1.500 | 0.216 | 0.051 | 0.024 | 0.112 | 0.871 | 27.300 | 38.300 | 36.000 | 0.737 | 26.893 | 28.134 |
| FT3D.fall | 17.831 | 41.057 | 48.640 | 1.341 | 1.033 | 0.171 | 0.209 | 1.641 | 28.503 | 38.656 | 7.000 | 0.137 | NA | 37.731 |
| FT3D.spring | 17.831 | 41.057 | 31.000 | 0.800 | 0.357 | 0.117 | 0.146 | 0.863 | 27.200 | 38.300 | 58.000 | 0.500 | 45.960 | 60.759 |
| FT3S.fall | 17.831 | 41.057 | 3.800 | 0.097 | 0.493 | 0.050 | 0.100 | 0.392 | 32.100 | 38.700 | 7.000 | 0.137 | NA | 44.028 |
| FT3S.spring | 17.831 | 41.057 | 1.700 | 0.182 | 0.115 | 0.010 | 0.096 | 0.376 | 27.400 | 38.800 | 58.000 | 0.500 | 41.828 | 42.803 |
| FT4D.fall | 17.292 | 40.757 | 40.400 | 1.890 | 4.327 | 0.425 | 0.100 | 4.585 | 29.500 | 38.700 | NA | NA | 36.766 | NA |
| FT4D.spring | 17.292 | 40.757 | 34.000 | 0.417 | 0.135 | 0.063 | 0.162 | 1.191 | 25.800 | 37.700 | 62.000 | 0.639 | 26.698 | 8.931 |
| FT4S.spring | 17.292 | 40.757 | 1.700 | 0.247 | 0.058 | 0.016 | 0.191 | 1.165 | 26.200 | 37.700 | 62.000 | 0.639 | 43.846 | 32.715 |
| FT5D.fall | 17.296 | 40.743 | 46.058 | 0.412 | 4.453 | 0.505 | 0.425 | 3.104 | 29.723 | 38.521 | 34.000 | 0.188 | 41.884 | 50.962 |
| FT5D.spring | 17.296 | 40.743 | 30.000 | 0.622 | 0.316 | 0.165 | 0.199 | 0.773 | 27.200 | 38.300 | 49.000 | 0.436 | 48.085 | 54.776 |
| FT5S.fall | 17.296 | 40.743 | 5.000 | 0.070 | 0.498 | 0.050 | 0.100 | 0.348 | 30.100 | 40.100 | 34.000 | 0.188 | 27.595 | 54.241 |
| FT5S.spring | 17.296 | 40.743 | 2.000 | 0.245 | 0.234 | 0.044 | 0.142 | 0.775 | 27.100 | 38.000 | 49.000 | 0.436 | 29.721 | 21.809 |
| FT6D.fall | 17.296 | 41.102 | 39.929 | 0.215 | 1.134 | 0.119 | 0.180 | 0.938 | 29.436 | 38.571 | 25.000 | 0.134 | 32.876 | 45.770 |
| FT6D.spring | 17.296 | 41.102 | 20.000 | 0.650 | 0.128 | 0.129 | 0.172 | 0.764 | 27.300 | 38.300 | NA | 0.359 | 43.846 | 48.291 |
| FT6S.fall | 17.296 | 41.102 | 2.319 | 0.215 | 0.772 | 0.050 | 0.100 | 0.436 | 32.132 | 38.820 | 25.000 | 0.134 | 28.875 | 47.729 |
| FT6S.spring | 17.296 | 41.102 | 1.800 | 0.280 | 0.070 | 0.055 | 0.126 | 0.557 | 27.400 | 38.100 | NA | 0.359 | 29.687 | 38.884 |
| FT8D-1.spring | 17.296 | 41.767 | 25.000 | 0.764 | 0.266 | 0.198 | 0.200 | 1.469 | 26.000 | 37.600 | 56.000 | 0.373 | 25.445 | 24.194 |
| FT8D.fall | 17.296 | 41.767 | 35.237 | 1.142 | 0.944 | 0.181 | 0.200 | 1.897 | 30.071 | 38.606 | 11.000 | 0.422 | 40.983 | 42.267 |
| FT8D.spring | 17.296 | 41.767 | 25.000 | 0.764 | 0.266 | 0.198 | 0.200 | 1.469 | 26.000 | 37.600 | 56.000 | 0.373 | 27.806 | 9.379 |
| FT8S.fall | 17.296 | 41.767 | 2.716 | 0.437 | 0.513 | 0.050 | 0.100 | 0.390 | 32.685 | 38.920 | 11.000 | 0.422 | 28.989 | 40.010 |
| JEC11D.fall | 17.297 | 42.104 | 35.797 | 5.070 | 2.482 | 0.755 | 0.361 | 3.053 | 30.324 | 38.677 | 9.000 | 0.456 | NA | 35.301 |
| JEC11S-2.fall | 17.297 | 42.104 | 2.788 | 5.070 | 1.399 | 0.075 | 0.214 | 0.979 | 32.958 | 38.818 | 9.000 | 0.456 | 28.473 | 28.290 |
| JEC11S.fall | 17.297 | 42.104 | 2.788 | 5.070 | 1.399 | 0.075 | 0.214 | 0.979 | 32.958 | 38.818 | 9.000 | 0.456 | 16.942 | 30.910 |
| JEC12D.fall | 17.263 | 42.302 | 8.500 | 0.400 | 2.436 | 1.079 | 0.333 | 4.429 | 33.400 | 39.100 | 10.000 | 0.327 | 14.445 | NA |
| JEC12S.fall | 17.263 | 42.302 | 2.884 | 0.942 | 0.325 | 0.050 | 0.100 | 1.256 | 33.112 | 38.931 | 10.000 | 0.327 | 35.974 | 29.643 |
| JEC14D.fall | 17.258 | 42.215 | 28.600 | 2.050 | NA | NA | NA | NA | 31.300 | 38.700 | 38.000 | 0.403 | NA | 26.673 |
| JEC14S.fall | 17.258 | 42.215 | 2.807 | 1.162 | 0.355 | 0.050 | 0.100 | 0.635 | 33.094 | 38.585 | 38.000 | 0.403 | 20.120 | 38.896 |
| JEC17S.spring | 17.186 | 42.246 | 1.000 | 0.465 | 0.191 | 0.080 | 0.142 | 1.256 | 27.300 | 37.900 | 48.000 | 0.527 | 47.368 | 51.796 |
| JEC1S.fall | 17.432 | 42.271 | 3.467 | 1.311 | 1.519 | 0.072 | 0.100 | 2.579 | 33.226 | 38.753 | 12.000 | 0.717 | 17.097 | 30.420 |
| JEC3D.fall | 17.432 | 42.194 | 23.500 | 0.730 | NA | NA | 0.100 | 0.511 | 32.800 | 39.100 | NA | 0.557 |  | 32.158 |
| JEC3S.fall | 17.432 | 42.194 | 3.004 | 0.677 | 0.245 | 0.050 | 0.100 | 2.528 | 32.969 | 38.132 | NA | 0.557 | 28.953 | 31.101 |
| JEC6D.fall | 17.397 | 42.200 | 25.868 | 1.179 | 0.359 | 0.097 | 0.107 | 0.632 | 31.922 | 38.782 | 10.000 | 0.708 | 39.598 | 37.818 |
| JEC6S.fall | 17.397 | 42.200 | 3.008 | 0.937 | 0.472 | 0.050 | 0.100 | 2.409 | 33.355 | 38.975 | 10.000 | 0.708 | NA | 32.328 |
| JN1D.spring | 21.591 | 38.996 | 60.280 | NA | NA | NA | NA | NA | 26.162 | 39.160 | NA | NA | NA | 24.137 |
| JN1S.spring | 21.591 | 38.996 | 5.250 | NA | NA | NA | NA | NA | 27.143 | 38.733 | NA | NA | NA | 23.384 |
| JN2D.spring | 21.591 | 39.020 | 59.760 | NA | NA | NA | NA | NA | 26.235 | 39.135 | NA | NA | NA | 35.223 |
| JN2S.spring | 21.591 | 39.020 | 5.400 | NA | NA | NA | NA | NA | 27.161 | 38.797 | NA | NA | NA | 33.626 |
| JN3D.spring | 21.591 | 39.045 | 59.700 | NA | NA | NA | NA | NA | 26.165 | 39.168 | NA | NA | NA | 35.753 |
| JN3S.spring | 21.591 | 39.045 | 5.650 | NA | NA | NA | NA | NA | 27.157 | 38.858 | NA | NA | NA | 32.716 |
| JN4D.spring | 21.591 | 39.069 | 60.600 | NA | NA | NA | NA | NA | 26.313 | 39.083 | NA | NA | NA | 24.398 |
| JN4S.spring | 21.591 | 39.069 | 7.010 | NA | NA | NA | NA | NA | 27.170 | 38.847 | NA | NA | NA | 30.742 |
| JN5D.spring | 21.609 | 39.093 | 80.760 | NA | NA | NA | NA | NA | 25.907 | 39.278 | NA | NA | NA | 38.309 |
| JN5S.spring | 21.609 | 39.093 | 4.960 | NA | NA | NA | NA | NA | 27.190 | 38.820 | NA | NA | NA | 35.580 |
| JN6D.spring | 21.599 | 39.096 | 58.070 | NA | NA | NA | NA | NA | 26.374 | 39.048 | NA | NA | NA | 34.634 |
| JN6S.spring | 21.599 | 39.096 | 5.080 | NA | NA | NA | NA | NA | 27.406 | 38.838 | NA | NA | NA | 27.772 |
| JN9D.spring | 21.572 | 39.100 | 62.000 | NA | NA | NA | NA | NA | 26.636 | 38.966 | NA | NA | NA | 5.344 |
| JN9S.spring | 21.572 | 39.100 | 6.670 | NA | NA | NA | NA | NA | 27.769 | 38.852 | NA | NA | NA | 27.926 |
| JS1S.spring | 21.342 | 38.996 | 5.090 | NA | NA | NA | NA | NA | 27.168 | 38.650 | NA | NA | NA | 34.324 |
| JS2D.spring | 21.341 | 39.021 | 85.050 | NA | NA | NA | NA | NA | 25.828 | 39.350 | NA | NA | NA | 44.251 |
| JS2S.spring | 21.341 | 39.021 | 4.980 | NA | NA | NA | NA | NA | 27.452 | 38.645 | NA | NA | NA | 21.824 |
| JS3D.spring | 21.342 | 39.059 | 30.180 | NA | NA | NA | NA | NA | 26.946 | 38.746 | NA | NA | NA | 37.068 |
| JS4D.spring | 21.342 | 39.084 | 44.870 | NA | NA | NA | NA | NA | 26.825 | 38.909 | NA | NA | NA | 39.408 |
| JS4S.spring | 21.342 | 39.084 | 5.160 | NA | NA | NA | NA | NA | 27.618 | 38.658 | NA | NA | NA | 32.132 |
| JS8D.spring | 21.306 | 39.088 | 61.810 | NA | NA | NA | NA | NA | 26.828 | 38.958 | NA | NA | NA | 38.073 |
| JS8S.spring | 21.306 | 39.088 | 5.170 | NA | NA | NA | NA | NA | 27.691 | 38.710 | NA | NA | NA | 34.089 |
| SR3D.spring | 18.974 | 39.541 | 63.000 | 0.369 | 0.473 | 0.313 | 0.173 | 1.375 | 27.400 | 38.600 | 37.000 | 0.242 | 54.358 | 46.371 |
| SR3S.spring | 18.974 | 39.541 | 5.000 | 0.136 | 0.053 | 0.045 | 0.225 | 1.305 | 26.800 | 37.900 | 37.000 | 0.242 | 46.334 | 41.998 |
| SR4D.spring | 17.737 | 40.358 | 53.000 | 0.646 | 0.497 | 0.359 | 0.209 | 1.360 | 27.700 | 39.000 | 38.000 | 0.590 | 34.821 | 37.618 |
| SR4S.spring | 17.737 | 40.358 | 1.500 | 0.176 | 0.115 | 0.060 | 0.103 | 0.585 | 27.100 | 38.200 | 38.000 | 0.590 | 28.307 | 49.203 |
| SR5D.spring | 16.696 | 41.196 | 26.000 | 0.637 | 0.124 | 0.073 | 0.175 | 1.608 | 26.100 | 37.600 | 46.000 | 0.421 | 48.205 | 41.258 |
| SR5S.spring | 16.696 | 41.196 | 3.000 | 0.303 | 0.122 | 0.019 | 0.173 | 1.575 | 26.300 | 37.500 | 46.000 | 0.421 | 20.090 | 11.720 |
| WA2S.fall | 25.888 | 36.496 | 5.000 | 0.079 | 0.430 | 0.050 | 0.100 | 0.812 | 30.670 | 40.138 | 10.000 | NA | 26.267 | 47.897 |
| WA3S.fall | 25.825 | 36.311 | 5.000 | 0.046 | 0.456 | 0.050 | 0.100 | 0.618 | 30.008 | 40.111 | 10.000 | NA | 22.076 | 45.286 |
| WA4S.fall | 25.760 | 36.131 | 5.000 | 0.058 | 0.485 | 0.050 | 0.100 | 0.468 | 29.707 | 39.755 | 9.000 | NA | 30.899 | 48.184 |

Supplementary Table 2: ANOVA and TukeyHSD results for nutrients and dissimilarity results.

|  |  | Eukaryote | | Prokaryote | |  |  |  |
| --- | --- | --- | --- | --- | --- | --- | --- | --- |
|  |  | UniFrac | | | |  |  |  |
|  |  | Unweighted | Weighted | Unweighted | Weighted | Nitrate | Phosphate | Silicate |
| Anova |  |  |  |  |  |  |  |  |
|  | F value | 338.9 | 473.4 | 105.8 | 92.28 | 9.338 | 10.32 | 10.79 |
|  | p | **<0.001** | **<0.001** | **<0.001** | **<0.001** | **<0.001** | **<0.001** | **<0.001** |
|  |  |  |  |  |  |  |  |  |
|  |  |  |  |  |  |  |  |  |
| TukeyHSD |  |  |  |  |  |  |  |  |
|  | North_Spring-North_Fall | **<0.001** | **<0.001** | **<0.001** | **0.001** | 0.926 | 0.733 | 0.625 |
|  | South_Fall-North_Fall | **<0.001** | **<0.001** | **<0.001** | **0.001** | **<0.001** | **<0.001** | **<0.001** |
|  | South_Spring-North_Fall | **<0.001** | **<0.001** | **<0.001** | **0.001** | 0.427 | **0.006** | 0.104 |
|  | South_Fall-North_Spring | 0.946 | **<0.001** | **<0.001** | 0.899 | **0.033** | **<0.001** | **0.005** |
|  | South_Spring-North_Spring | **<0.001** | **0.034** | **<0.001** | **0.049** | 0.283 | **0.002** | 0.839 |
|  | South_Spring-South_Fall | **<0.001** | **<0.001** | **<0.001** | 0.087 | **<0.001** | 0.956 | **0.047** |

Supplementary Table 3: Pairwise comparisons of community structure and composition.

|  |  | **Eukaryotes unweighted** | | |
| --- | --- | --- | --- | --- |
| Source | df | MS | Pseudo-F | P(perm) |
| Re | 1 | 1.7264 | 13.021 | 0.0001 |
| De | 1 | 1.3371 | 10.085 | 0.0001 |
| Se | 1 | 2.2228 | 16.765 | 0.0001 |
| RexDe | 1 | 0.33357 | 2.5159 | 0.0005 |
| RexSe | 1 | 0.87857 | 6.6266 | 0.0001 |
| DexSe | 1 | 0.38846 | 2.93 | 0.0003 |
| RexDexSe | 1 | 0.21122 | 1.5931 | 0.024 |
| Res | 120 | 0.13258 |  |  |
| Total | 127 |  |  |  |
|  |  |  |  |  |
| Pairwise tests |  |  |  |  |
|  |  | Term 'RexDexSe' for pairs of levels of factor 'Region' |  |  |
|  |  | Within level 'DCM' of factor 'Depth' |  |  |
|  |  | Within level 'Spring' of factor 'Season' |  |  |
|  |  |  | North ≠ South |  |
|  |  | Within level 'DCM' of factor 'Depth' |  |  |
|  |  | Within level 'Fall' of factor 'Season' |  |  |
|  |  |  | North ≠ South |  |
|  |  | Within level 'Surface' of factor 'Depth' |  |  |
|  |  | Within level 'Spring' of factor 'Season' |  |  |
|  |  |  | North ≠ South |  |
|  |  | Within level 'Surface' of factor 'Depth' |  |  |
|  |  | Within level 'Fall' of factor 'Season' |  |  |
|  |  |  | North ≠ South |  |
|  |  | Term 'RexDexSe' for pairs of levels of factor 'Depth' |  |  |
|  |  | Within level 'North' of factor 'Region' |  |  |
|  |  | Within level 'Spring' of factor 'Season' |  |  |
|  |  |  | Surface ≠ DCM |  |
|  |  | Within level 'North' of factor 'Region' |  |  |
|  |  | Within level 'Fall' of factor 'Season' |  |  |
|  |  |  | Surface ≠ DCM |  |
|  |  | Within level 'South' of factor 'Region' |  |  |
|  |  | Within level 'Spring' of factor 'Season' |  |  |
|  |  |  | Surface = DCM |  |
|  |  | Within level 'South' of factor 'Region' |  |  |
|  |  | Within level 'Fall' of factor 'Season' |  |  |
|  |  |  | Surface ≠ DCM |  |
|  |  | Term 'RexDexSe' for pairs of levels of factor 'Season' |  |  |
|  |  | Within level 'North' of factor 'Region' |  |  |
|  |  | Within level 'DCM' of factor 'Depth' |  |  |
|  |  |  | Spring ≠ Fall |  |
|  |  | Within level 'North' of factor 'Region' |  |  |
|  |  | Within level 'Surface' of factor 'Depth' |  |  |
|  |  |  | Spring ≠ Fall |  |
|  |  | Within level 'South' of factor 'Region' |  |  |
|  |  | Within level 'DCM' of factor 'Depth' |  |  |
|  |  |  | Spring ≠ Fall |  |
|  |  | Within level 'South' of factor 'Region' |  |  |
|  |  | Within level 'Surface' of factor 'Depth' |  |  |
|  |  |  | Spring ≠ Fall |  |
|  |  |  |  |  |
|  |  |  |  |  |
| Source | df | **Eukaryotes weighted** |  |  |
| Re | 1 | MS | Pseudo-F | P(perm) |
| De | 1 | 0.9754 | 12.762 | 0.0001 |
| Se | 1 | 0.95108 | 12.444 | 0.0001 |
| RexDe | 1 | 1.1389 | 14.902 | 0.0001 |
| RexSe | 1 | 0.18714 | 2.4485 | 0.0128 |
| DexSe | 1 | 1.1995 | 15.694 | 0.0001 |
| RexDexSe | 1 | 0.21066 | 2.7562 | 0.0048 |
| Res | 120 | 0.17764 | 2.3242 | 0.0153 |
| Total | 127 | 0.076431 |  |  |
|  |  |  |  |  |
| Pairwise tests |  |  |  |  |
|  |  |  |  |  |
|  |  | Term 'RexDexSe' for pairs of levels of factor 'Region' |  |  |
|  |  | Within level 'DCM' of factor 'Depth' |  |  |
|  |  | Within level 'Spring' of factor 'Season' |  |  |
|  |  |  | North ≠ South |  |
|  |  | Within level 'DCM' of factor 'Depth' |  |  |
|  |  | Within level 'Fall' of factor 'Season' |  |  |
|  |  |  | North ≠ South |  |
|  |  | Within level 'Surface' of factor 'Depth' |  |  |
|  |  | Within level 'Spring' of factor 'Season' |  |  |
|  |  |  | North ≠ South |  |
|  |  | Within level 'Surface' of factor 'Depth' |  |  |
|  |  | Within level 'Fall' of factor 'Season' |  |  |
|  |  |  | North ≠ South |  |
|  |  | Term 'RexDexSe' for pairs of levels of factor 'Depth' |  |  |
|  |  | Within level 'North' of factor 'Region' |  |  |
|  |  | Within level 'Spring' of factor 'Season' |  |  |
|  |  |  | Surface = DCM |  |
|  |  | Within level 'North' of factor 'Region' |  |  |
|  |  | Within level 'Fall' of factor 'Season' |  |  |
|  |  |  | Surface ≠ DCM |  |
|  |  | Within level 'South' of factor 'Region' |  |  |
|  |  | Within level 'Spring' of factor 'Season' |  |  |
|  |  |  | Surface = DCM |  |
|  |  | Within level 'South' of factor 'Region' |  |  |
|  |  | Within level 'Fall' of factor 'Season' |  |  |
|  |  |  | Surface ≠ DCM |  |
|  |  | Term 'RexDexSe' for pairs of levels of factor 'Season' |  |  |
|  |  | Within level 'North' of factor 'Region' |  |  |
|  |  | Within level 'DCM' of factor 'Depth' |  |  |
|  |  |  | Spring ≠ Fall |  |
|  |  | Within level 'North' of factor 'Region' |  |  |
|  |  | Within level 'Surface' of factor 'Depth' |  |  |
|  |  |  | Spring ≠ Fall |  |
|  |  | Within level 'South' of factor 'Region' |  |  |
|  |  | Within level 'DCM' of factor 'Depth' |  |  |
|  |  |  | Spring ≠ Fall |  |
|  |  | Within level 'South' of factor 'Region' |  |  |
|  |  | Within level 'Surface' of factor 'Depth' |  |  |
|  |  |  | Spring ≠ Fall |  |
|  |  |  |  |  |
|  |  |  |  |  |
|  |  |  |  |  |
|  |  | **Prokaryotes unweighted** | | |
| Source | df | MS | Pseudo-F | P(perm) |
| Re | 1 | 0.97303 | 7.9086 | 0.0001 |
| De | 1 | 1.3421 | 10.908 | 0.0001 |
| Se | 1 | 1.3568 | 11.028 | 0.0001 |
| RexDe | 1 | 0.25778 | 2.0952 | 0.0043 |
| RexSe | 1 | 0.42368 | 3.4436 | 0.0001 |
| DexSe | 1 | 0.36989 | 3.0064 | 0.0001 |
| RexDexSe | 1 | 0.1478 | 1.2013 | 0.1689 |
| Res | 113 | 0.12303 |  |  |
| Total | 120 |  |  |  |
|  |  |  |  |  |
| Pairwise tests |  |  |  |  |
|  |  | Term 'RexDe' for pairs of levels of factor 'Region' |  |  |
|  |  | Within level 'DCM' of factor 'Depth' |  |  |
|  |  |  | North ≠ South |  |
|  |  | Within level 'Surface' of factor 'Depth' |  |  |
|  |  |  | North ≠ South |  |
|  |  | Term 'RexDe' for pairs of levels of factor 'Depth' |  |  |
|  |  | Within level 'North' of factor 'Region' |  |  |
|  |  |  | Surface ≠ DCM |  |
|  |  | Within level 'South' of factor 'Region' |  |  |
|  |  |  | Surface ≠ DCM |  |
|  |  | Term 'RexSe' for pairs of levels of factor 'Region' |  |  |
|  |  | Within level 'Spring' of factor 'Season' |  |  |
|  |  |  | North ≠ South |  |
|  |  | Within level 'Fall' of factor 'Season' |  |  |
|  |  |  | North ≠ South |  |
|  |  | Term 'RexSe' for pairs of levels of factor 'Season' |  |  |
|  |  | Within level 'North' of factor 'Region' |  |  |
|  |  |  | Spring ≠ Fall |  |
|  |  | Within level 'South' of factor 'Region' |  |  |
|  |  |  | Spring ≠ Fall |  |
|  |  | Term 'DexSe' for pairs of levels of factor 'Season' |  |  |
|  |  | Within level 'DCM' of factor 'Depth' |  |  |
|  |  |  | Spring ≠ Fall |  |
|  |  | Within level 'Surface' of factor 'Depth' |  |  |
|  |  |  | Spring ≠ Fall |  |
|  |  | Term 'DexSe' for pairs of levels of factor 'Depth' |  |  |
|  |  | Within level 'Spring' of factor 'Season' |  |  |
|  |  |  | Surface ≠ DCM |  |
|  |  | Within level 'Fall' of factor 'Season' |  |  |
|  |  |  | Surface ≠ DCM |  |
|  |  |  |  |  |
|  |  |  |  |  |
|  |  |  |  |  |
|  |  | **Prokaryotes weighted** | | |
| Source | df | MS | Pseudo-F | P(perm) |
| Re | 1 | 0.10568 | 8.9269 | 0.0001 |
| De | 1 | 0.18995 | 16.045 | 0.0001 |
| Se | 1 | 0.84953 | 71.759 | 0.0001 |
| RexDe | 1 | 0.023473 | 1.9827 | 0.0685 |
| RexSe | 1 | 0.05288 | 4.4667 | 0.0004 |
| DexSe | 1 | 0.076144 | 6.4318 | 0.0001 |
| RexDexSe | 1 | 0.011634 | 0.98271 | 0.4249 |
| Res | 113 | 0.011839 |  |  |
| Total | 120 |  |  |  |
|  |  |  |  |  |
| Pairwise tests |  |  |  |  |
|  |  | Term 'RexSe' for pairs of levels of factor 'Region' |  |  |
|  |  | Within level 'Spring' of factor 'Season' |  |  |
|  |  |  | North ≠ South |  |
|  |  | Within level 'Fall' of factor 'Season' |  |  |
|  |  |  | North ≠ South |  |
|  |  | Term 'RexSe' for pairs of levels of factor 'Season' |  |  |
|  |  | Within level 'North' of factor 'Region' |  |  |
|  |  |  | Spring ≠ Fall |  |
|  |  | Within level 'South' of factor 'Region' |  |  |
|  |  |  | Spring ≠ Fall |  |
|  |  | Term 'DexSe' for pairs of levels of factor 'Depth' |  |  |
|  |  | Within level 'Spring' of factor 'Season' |  |  |
|  |  |  | Surface ≠ DCM |  |
|  |  | Within level 'Fall' of factor 'Season' |  |  |
|  |  |  | Surface ≠ DCM |  |
|  |  | Term 'DexSe' for pairs of levels of factor 'Season' |  |  |
|  |  | Within level 'DCM' of factor 'Depth' |  |  |
|  |  |  | Spring ≠ Fall |  |
|  |  | Within level 'Surface' of factor 'Depth' |  |  |
|  |  |  | Spring ≠ Fall |  |
